# Nanostructure and dynamics of N-truncated copper amyloid-β peptides from advanced X-ray absorption fine structure

**DOI:** 10.1107/S2052252524001830

**Published:** 2024-04-11

**Authors:** Ruwini S. K. Ekanayake, Victor A. Streltsov, Stephen P. Best, Christopher T. Chantler

**Affiliations:** a School of Physics, University of Melbourne, Australia; b The Florey Institute of Neuroscience and Mental Health, University of Melbourne, Australia; c School of Chemistry, University of Melbourne, Australia; Australian Nuclear Science and Technology Organisation and University of Wollongong, Australia

**Keywords:** N-truncated copper amyloid-β peptides, X-ray absorption fine structure, radiation damage, nanostructure, XAS electrochemical cells, binding motifs, redox cycles

## Abstract

An X-ray absorption spectroscopy (XAS) electrochemical cell was used to collect high-quality XAS measurements of N-truncated Cu:amyloid-β (Cu:Aβ) samples under near-physiological conditions. N-truncated Cu:Aβ peptide complexes contribute to oxidative stress and neurotoxicity in Alzheimer’s patients’ brains. However, the redox properties of copper in different Aβ peptide sequences are inconsistent. Therefore, the geometry of binding sites for the copper binding in Aβ_4–8/12/16_ was determined using novel advanced extended X-ray absorption fine structure (EXAFS) analysis. This enables these peptides to perform redox cycles in a manner that might produce toxicity in human brains. Fluorescence XAS measurements were corrected for systematic errors including defective-pixel data, monochromator glitches and dispersion of pixel spectra. Experimental uncertainties at each data point were measured explicitly from the point-wise variance of corrected pixel measurements. The copper-binding environments of Aβ_4–8/12/16_ were precisely determined by fitting XAS measurements with propagated experimental uncertainties, advanced analysis and hypothesis testing, providing a mechanism to pursue many similarly complex questions in bioscience. The low-temperature XAS measurements here determine that Cu^II^ is bound to the first amino acids in the high-affinity amino-terminal copper and nickel (ATCUN) binding motif with an oxygen in a tetragonal pyramid geometry in the Aβ_4–8/12/16_ peptides. Room-temperature XAS electrochemical-cell measurements observe metal reduction in the Aβ_4–16_ peptide. Robust investigations of XAS provide structural details of Cu^II^ binding with a very different *bis*-His motif and a water oxygen in a quasi-tetrahedral geometry. Oxidized XAS measurements of Aβ_4–12/16_ imply that both Cu^II^ and Cu^III^ are accommodated in an ATCUN-like binding site. Hypotheses for these Cu^I^, Cu^II^ and Cu^III^ geometries were proven and disproven using the novel data and statistical analysis including *F* tests. Structural parameters were determined with an accuracy some tenfold better than literature claims of past work. A new protocol was also developed using EXAFS data analysis for monitoring radiation damage. This gives a template for advanced analysis of complex biosystems.

## Abstract 2

An X-ray absorption spectroscopy electrochemical cell was used to collect high-quality X-ray absorption spectroscopy measurements of N-truncated Cu:amyloid-β (Cu:Aβ) samples under near-physiological conditions. The geometry of binding sites for the copper binding in Aβ_4–8/12/16_ and the ability of these peptides to perform redox cycles in a manner that might produce toxicity in human brains were determined.

## 1. Copper binding with N-truncated amyloid-β peptides and links with Alzheimer’s disease

Alzheimer’s disease (AD) is a common progressive brain disorder that develops into irreversible dementia (Holtzman *et al.*, 2011 ▸). AD is characterized by association with the existence of amyloid plaques in the human brain, which mainly consist of amyloid-β (Aβ) peptides. Aggregation of amyloid cascades combined with metal-ion oxidation lead to toxic functions, including generation of reactive oxygen species (ROS) (Hureau, 2012 ▸). The toxicity relating to ROS is produced by transition metal ions such as Cu, Zn and Fe bound to the Aβ peptide (Smith *et al.*, 2000 ▸; Barnham *et al.*, 2004 ▸; Ganguly *et al.*, 2017 ▸; Drew, 2017 ▸). Furthermore, N- and C-terminal heterogeneity was reported in early protein studies of amyloid plaque cores (APCs) in AD brains (Masters *et al.*, 1985*b* ▸) and *in vitro* experiments (Pike *et al.*, 1995 ▸). The majority of APCs consist of Aβ sequences starting at position 4, the phenylalanine (Phe4) residue (Masters *et al.*, 1985*a* ▸,*b* ▸). Interestingly, metal-bound N-truncated Aβ peptides create a higher neurotoxicity (McLean *et al.*, 1999 ▸; Cheignon *et al.*, 2018 ▸) than full-length Aβ in patients’ brains (Dietrich *et al.*, 2018 ▸; Dunys *et al.*, 2018 ▸; Cabrera *et al.*, 2018 ▸). A better understanding of the redox behaviour of Cu-bound N-truncated peptides and the toxicity arising from radicals is crucial for pathogenesis.

Different coordination spheres of Cu-binding geometry in Aβ peptides and in the affinity of the metal have been reported (Streltsov *et al.*, 2008 ▸; Faller & Hureau, 2009 ▸; Summers *et al.*, 2019 ▸; Abelein *et al.*, 2022 ▸). Cu^II^ binding may involve three N and one O (Huang *et al.*, 1999 ▸; Wang & Hanson, 1995 ▸). The three N coordinations are associated with three His residues; the fourth O coordination could arise from water, carboxyl or hydroxyl side chains, or a phosphate buffer in Aβ_1–42/28_ peptides (Curtain *et al.*, 2001 ▸; Drew *et al.*, 2009*a* ▸).

N-truncated Aβ_4–*y*_ peptides may accommodate a high-affinity amino-terminal copper and nickel (ATCUN; H_2_N-*X*-*X*-His) motif in their structure due to the phenylalanine, arginine and histidine (F4R5H6) residues at the end of the peptide (Harford & Sarkar, 1997 ▸; Mital *et al.*, 2015 ▸; Bossak-Ahmad *et al.*, 2019 ▸; Esmieu *et al.*, 2021 ▸). The motif accommodates a chelate ring including terminal amine-Phe4, deprotonated backbone amides from Arg5 and His6, and His6-imidazole nitrogen donor atoms (Harford & Sarkar, 1997 ▸; Sóvágó & Ősz, 2006 ▸). Similar involvement of nitrogens in Cu^II^ binding was identified (Shearer *et al.*, 1967 ▸) in serum albumin. ATCUN-chelated binding coordination through tetradentate ligands has also been considered (Camerman *et al.*, 1976 ▸). Hureau *et al.* (2011 ▸) proposed high-affinity Cu^II^ chelating coordination geometries and binding of a water molecule in the apical position for Cu^II^GHK and Cu^II^DAHK complexes using X-ray analysis. Mital *et al.* (2015 ▸) suggested an involvement of Cu^II^ with the ATCUN motif in N-truncated Aβ_4–16_ peptides.

Karr *et al.* (2005 ▸) suggested that Cu^II^ binding has no involvement with Tyr10 using electron paramagnetic resonance (EPR) spectra, whereas Stellato *et al.* (2006 ▸) suggested Cu binding to the oxygen atom in the Tyr10 hydroxyl group from X-ray absorption near-edge structure (XANES) and X-ray absorption fine structure (XAFS) analysis. Mital *et al.* (2015 ▸) performed the pH dependance of the Cu^II^ binding in N-truncated Aβ and reported a pH value of about 10 for Tyr10. Streltsov *et al.* (2008 ▸) suggested Cu^II^ binding in the peptide across His6/13/14, and with Asp1 or Glu11 residues in the full-length peptides. Karr *et al.* (2005 ▸) claimed that Cu^II^ binding is involved only with His6/13 using different-length Aβ peptides and EPR spectroscopy. Involvement of Tyr10 and Glu11 residues in Cu binding has been a controversial and much disputed subject.

Cu binding with the ATCUN followed by a *bis*-His motif is illustrated for protein structures such as transmembrane Cu transport protein (CTR1) (Pushie *et al.*, 2015 ▸). CTR1 and the Hst5 antimicrobial peptide (AMP) separate the *bis*-His motif from the ATCUN motif compared with N-truncated Aβ sequences (Tay *et al.*, 2009 ▸).

Best *et al.* (2016 ▸) introduced a flow cell to the standard fluorescence X-ray absorption spectroscopy (XAS) experimental setup to obtain XAS measurements of any biological compound such as organometals, proteins and catalysts with minimal radiation damage.

The involvement of nitrogens and oxygens for the coordination Cu^I^ in the Aβ peptides was suggested (Himes *et al.*, 2007 ▸, 2008 ▸; Raffa *et al.*, 2007 ▸; Shearer & Szalai, 2008 ▸; Hureau *et al.*, 2009 ▸; Balland & Hureau, 2010 ▸; Furlan *et al.*, 2010 ▸). Pushie *et al.* (2015 ▸) proposed quasi-tetrahedral four-coordinated Cu^I^ binding involving two imidazole N from *bis*-His, one S and one backbone carbonyl O. They claimed that their proposed model is more probable than the two-coordinated *bis*-His Cu^I^ structures. Streltsov *et al.* (2018 ▸) suggested a quasi-tetrahedral geometry for Cu^I^ binding in N-truncated Aβ_4–16_ using extended XAFS (EXAFS) analysis. The reported binding geometries are controversial, and there is no general agreement on Cu^I^ coordination geometry in peptides. Therefore, it is important to investigate Cu-binding coordination in different-length Aβ_4–*y*_ peptides.

## 2. Advanced analysis towards establishing the geometry of Cu-binding sites of N-truncated amyloid-β and refining precise structural parameters

In this article, we introduce advanced data and statistical analysis to a complex biosystem to determine molecular parameters, nanostructure and local environment to a much higher accuracy than otherwise possible with standard approaches. XAS is an ideal element-selective tool to investigate many biological samples, organometals and metal peptides, provides high-resolution nanostructural information, and is suitable for sensitive samples. Current XAS uses ‘goodness of fit’ measures, but without incorporating uncertainties derived from the standard deviations of experimental measurements (O’Day *et al.*, 1994 ▸; Filipponi & Di Cicco, 1995 ▸; Filipponi, 1995 ▸). A major issue is the propagation of uncertainty from experimental systematics in XAS (Chantler *et al.*, 1999 ▸, 2012 ▸; Chantler, 2009 ▸; Schalken & Chantler, 2018 ▸; Trevorah *et al.*, 2019 ▸).

This enables us to perform much more rigorous assessments and indeed carry out quantitative hypothesis testing of alternate bonding and novel bonding sites.

Herein, we establish the geometry of binding sites for key copper binding in N-truncated Aβ_4–*y*_ at low temperatures, and separately under near-physiological conditions. Aβ_4–8_, Aβ_4–12_ and Aβ_4–16_ (F4RHDS8GYEV12HHQK16) sequences of N-truncated Aβ peptides were investigated, based on their enhanced solubility relative to the full-length wild-type Aβ_1/4–42_ at low and room temperature. Aβ_4–16_ accommodates His6, His13 and His14 in its structure, whereas the shorter peptides only accommodate His6 in their structures. The shortest peptide does not include the tyrosine and glutamine residues in its sequence, allowing an investigation of the involvement of the absent residues in copper binding. Streltsov *et al.* (2018 ▸) performed preliminary analysis for identifying the geometry of Cu-binding sites in Cu-bound N-truncated Aβ_4–*y*_. In that study, conventional analysis used interpolated data points to generate EXAFS oscillations. In this work, data were analysed avoiding any interpolation and loss of information, to get more insightful results for hypothesis testing. Interpolations of experimental data on to a regularly spaced grid in *k* space would distort experimental values, information content, point density and experimental uncertainties (Schalken & Chantler, 2018 ▸). Furthermore, in this work we avoid guessing a constant data uncertainty, as in conventional analysis, and instead measure the self-consistency and data uncertainty by the reproducibility of data. More importantly, we can then use this to distinguish hypotheses with quantitative statistical measures including *F* tests.

We developed novel XAS under *in situ* electrochemical control (XAS-EC) (Streltsov *et al.*, 2018 ▸) to explore the redox properties of different-length Cu^II^-bound N-truncated Aβ peptides. Here, we determine the ability of Cu-bound N-truncated Aβ_4–8/12/16_ peptides to perform redox cycles in a manner that might produce ROS, facilitating oxidative damage under physiological conditions. To be explicit, XAS-EC is a novel development of this and our recent work, but this novel experimental methodology is not directly related to the X-ray extended range technique (Chantler *et al.*, 2001 ▸; Sier *et al.*, 2020 ▸; Ekanayake *et al.*, 2021 ▸; Chantler, 2022 ▸) or formally to the hybrid methodology (Schalken & Chantler, 2018 ▸; John *et al.*, 2023 ▸; Best & Chantler, 2022 ▸). Rather, key developments herein lie in data treatment and analysis.

This work also details the quality control of Aβ XAS measurements. As a core starting point, we propagated experimental uncertainties of measurements from point-wise variance of experimental measurements and experimental systematics. Systematic corrections are incorporated in uncertainty calculations to determine better uncertainties in fitted parameters. Error analysis based on the measured experimental uncertainties corresponding to experimental systematics and noise is limited in XAFS data analysis, and the absence of this can result in unreliable structural insight.

Here, we also determine the precise values of nano­structural bond lengths and thermal parameters from novel advanced EXAFS analysis.

An absolute determination of a structure for hypothesis testing was carried out using *eFEFFit* (Smale *et al.*, 2006 ▸; Schalken & Chantler, 2018 ▸), XAFS analysis, *FEFF6* (Zabinsky *et al.*, 1995 ▸) and *FEFF8* (Ankudinov *et al.*, 2003 ▸) for propagated uncertainties. We consider, for the first time, quantitative spectroscopic analysis of the coordination chemistry of Cu^II^, Cu^I^ and Cu^III^. Our initial investigations of the structural parameters of Cu-binding sites in Aβ_4–*y*_ (Streltsov *et al.*, 2018 ▸) did not include propagated uncertainties in the refinements. Estimated uncertainties were incorporated in the goodness-of-fit measurements. These uncertainties could then be under- or over-estimated due to contributions of noise, which can lead to limitations in hypothesis testing in structural refinement.

In this study, we also develop a new approach to monitor radiation damage that incorporates fits of individual scans of the Aβ_4–*y*_ peptides. The average of the repeated scans of Cu-bound N-truncated Aβ_4–*y*_ was refined in our initial investigations of the geometry of Cu-binding sites in the Aβ_4–*y*_ peptides (Streltsov *et al.*, 2018 ▸). If the photodamage is not properly identified, EXAFS analysis will be skewed, and therefore, the refined structural parameter will provide misleading information (Ekanayake *et al.*, 2024 ▸; Streltsov *et al.*, 2018 ▸).

## 3. Experimental methods

XAS at low temperatures and XAS-EC at room temperature were performed at the XAS beamline of the Australian Synchrotron (Streltsov *et al.*, 2018 ▸). The development of our experimental setup enables accurate XAS measurements of Cu-bound Aβ samples at ambient temperatures. The quality of spectra can be monitored and controlled during the data collection. We herein addressed systematic issues including defective-pixel exclusion, dead-time correction, deglitching, data truncation, detector inefficiency, data flattening and radiation damage, developed in detail by Ekanayake *et al.* (2024 ▸). Characteristic features of Cu^I^:Aβ_1–16_ were investigated by measuring XANES spectra with the potential stepped from −0.25 to −0.65 V (0.05 V, −0.05 V, −0.45 V, −0.65 V). The reduction of Cu^II^:Aβ_4–16_ to Cu^I^:Aβ_4–16_ was investigated by obtaining XANES-EC spectra at different reducing potentials from −0.15 to −0.45 V (−0.15 V, −0.25 V, −0.35 V, −0.45 V). The generation of oxidized products of Cu^II^:Aβ_4–8/16/12_ peptides was investigated at an oxidative potential of 0.95–1.35 V.

## 4. Cu^II^-binding ligands in N-truncated Aβ_4–8/12/16_ peptides from XAS

### 4.1. Identification of Cu^II^-binding ligands from XANES

**Figure 1:**
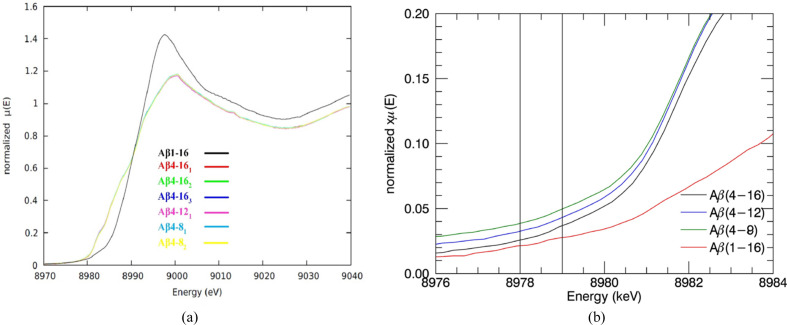
(*a*) XANES pre-edge spectra of 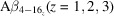 , 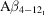 and 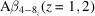 peptides highlighting the region relating to the 1*s*–3*d* transition. These three shorter peptides appear to have the same structure for copper binding. There appears to be no radiation photoreduction during the collection of data. By comparing these 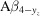 (*y* = 8, 12, 16 and *z* = 1, 2, 3) spectra with the spectrum of Aβ_1–16_ peptide, it is clear that these Aβ_4–*y*_ peptides do not have a structure similar to Aβ_1–16_ for copper binding. (*b*) The expanded pre-edge region of Aβ_1–16_ peptide showing a weak pre-edge peak at 8978 eV and Aβ_4–16/12/8_ peptides showing possible weak pre-edge peaks at 8979 eV. The (forbidden) 1*s*–3*d* region is affected by geometry and, for example, dihedral angles.

XANES spectra of N-truncated Cu^II^:Aβ_4–8/12/16_ and Cu^II^:Aβ_1–16_ peptides were investigated to compare apparent nanostructure prior to detailed XAFS analysis. The XANES spectra of the low-temperature XAS of Cu^II^:Aβ_4–8/12/16_ are virtually identical [Fig. 1 ▸(*a*)], suggesting identical Cu^II^-binding geometry for the three peptides, especially compared with the Cu^II^:Aβ_1–16_ spectrum.

Cu^II^:Aβ_4–8_ has neither Tyr10, Glu11 nor histidines (His13 or His14) in its structure. The consistency of spectra between all the Cu^II^:Aβ_4–8/12/16_ datasets suggests the same Cu^II^-binding ligands for all three peptides. Therefore, it must be dominated by the Cu^II^:Aβ_4–8_ peptides.

The spectra of Cu^II^:Aβ_1–16_ appear equivalent to previously measured spectra under similar conditions (Streltsov *et al.*, 2008 ▸), which suggested that the Cu^II^-binding site of Aβ_1–16_ involved either carboxylate O (Tyr10, Glu11) or histidine N atom (His13 or His14) coordination, but not the first three residues. The Cu^II^:Aβ_1–16_ spectrum is noticeably different from Cu^II^:Aβ_4–8/12/16_, suggesting a specific and different Cu^II^ -binding form. The Aβ_1–16_ Cu^II^ site has previously been shown to be highly pleomorphic and suggested to involve from one to all three histidine residues: H6H13H14 (Karr *et al.*, 2005 ▸; Drew *et al.*, 2009*b* ▸; Streltsov *et al.*, 2008 ▸; Yu *et al.*, 2008 ▸; Cheignon *et al.*, 2017 ▸). The current XANES data neither prove nor indicate any pleomorphism.

These identical characteristics of XANES Cu^II^:Aβ_4–8/12/16_ spectra justify the suggestion of some common site for the Cu^II^-binding geometry (Camerman *et al.*, 1976 ▸; Hureau *et al.*, 2011 ▸; Mital *et al.*, 2015 ▸; Bossak-Ahmad *et al.*, 2019 ▸) and prove the non-involvement of residues beyond the eighth peptide.

A very weak pre-edge peak at 8978 eV for Cu^II^:Aβ_1–16_ and a very weak feature at 8979 eV (Streltsov *et al.*, 2008 ▸, 2018 ▸; Pratesi *et al.*, 2012 ▸) for Cu^II^:Aβ_4–16_ [Fig. 1 ▸(*b*)] have been linked to the 1*s*–3*d* electric dipole forbidden transition of Cu^II^. A higher pre-edge intensity is usually observed for Cu^II^:Aβ_1–16_ with the increase of dihedral angles between ligands (Sano *et al.*, 1992 ▸). In the ATCUN-type Cu^II^:DAHK peptide, the N-terminal fragment of the human serum albumin has a consistent behaviour (Hureau *et al.*, 2011 ▸) in the pre-edge features at 8987 and 8988 eV, relating to the 1*s*–4*s* transition or 1*s*–4*p* transitions. This significant variation of intensity is probably related to the geometry of the ligands (Strange *et al.*, 1990 ▸; Streltsov *et al.*, 2008 ▸). It can also be affected by the spectral resolution (divergence, bandwidth, slit size, polarization).

The identical XANES spectra confirmed that neither Tyr10, Glu11 nor histidines (His13 or His14) are bound to the Cu. In these two proposed cases, the Cu^II^:Aβ_4–8_ structure would be very different from that of Cu^II^:Aβ_4–12/16_. Thus, the Cu^II^-binding site of all Aβ_4–8/12/16_ peptides appears to not involve either carboxylate O (Tyr10, Glu11) or histidine N atom (His13 or His14) coordination, and is instead formed by the first three residues. This limits the range of any possible proposed pleomorphism.

Streltsov *et al.* (2018 ▸) initially suggested the geometry by generating XANES and an interpolated grid using standard XAS analysis software. Important features in the pre-edge region, point density, and valuable information about the coordination geometry at different temperatures and under different experimental conditions are distorted when experimental data are interpolated into a regularly spaced grid. In this study, data processing yields more insightful results for hypothesis testing. The improvement of spectra and data processing primarily confirms this conclusion based on XANES, but a clear quantitative statistical conclusion is required from the following data section.

### 4.2. Structure determination of the Cu^II^-binding site in Aβ_4–8/12/16_ peptides from advanced analysis of low-temperature EXAFS

**Figure 2:**
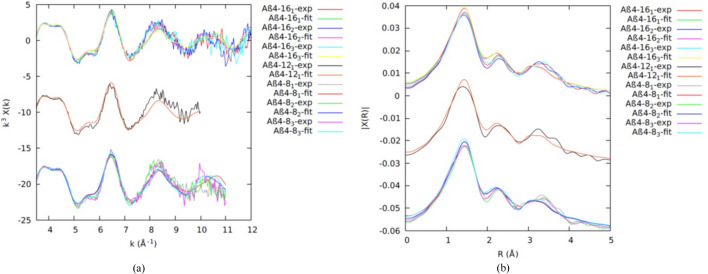
Individual EXAFS spectra of Cu^II^ 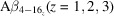 , Cu^II^ 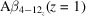 and Cu^II^ 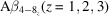 and their fits in (*a*) *k* space and (*b*) *R* space. Fits are generated from experimental data and propagated uncertainties using *eFEFFit* XAFS analysis. Some measurements were truncated at higher *k* due to statistical noise. The fitted individual structures are highly consonant within noise and within estimated uncertainty. The data are obviously limited by noise for the ranges, so the fits should also be; but the fits remain detailed and accurate. The transforms are more accurate and more self-similar than Streltsov *et al.* (2018 ▸). Hence, the accuracy of the results and the significance of the conclusions are dramatically improved.

Fig. 2 ▸ presents the data collected for the 4–*y* peptides, with repeated measurements. The data are self-similar, both in *k* and *R* space, and the resulting model fits all data well, within uncertainty, and with parameters that are consistent within uncertainty. Again, this argues for a common model, in a much more conclusive manner than the XANES evidence, albeit, at this stage, qualitative.

The spectroscopy appears very similar for each of the three Cu^II^Aβ_4–8/12/16_ peptide fragments. The fits of the model to individual EXAFS in *k* and *R* space (Fig. 2 ▸), and the resulting structural parameters (Table 1 ▸), are in good agreement within the uncertainty. The absolute uncertainties for the refined structural parameters use the propagated systematic data uncertainties.

The independent fitting parameters were: an energy offset of the energy threshold (δ*E*_0_), the amplitude reduction factor ( 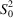 ), two independent thermal parameters for the axial water and for the third- and outer-shell neighbours ( 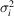 ), and ten independent radial adjustment distances. Eight restraint functions were also included to maintain reasonable parameters for 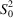 , 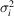 and five bond-length estimates. Additionally, two thermal parameters 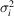 were fixed for the nearest-neighbour nitrogens (0.001 Å^2^) and for the second shell (0.00105 Å^2^).

Incorporation of multiple scattering and mean-square disorder for multiple scattering paths is essential (O’Day *et al.*, 1994 ▸; Ressler *et al.*, 1999 ▸). Multiple scattering contributions with greater than 10% relative predicted amplitude with triple scattering paths with four legs were included in the refinements. In general, the values of the shift in radial position of a peptide unit were tied to that of its nearest neighbour, and the corresponding 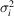 for that atom and in turn for relevant scattering paths including that atom was tied to that of the free parameters.

Consider for a moment the use of standard XAFS analysis, especially with the restricted *k* range presented, and with no simultaneously fitted range in *R* space. Then the traditional formula defines 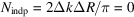 and 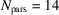 , and the usual metric using the so-called ‘Nyquist criterion’ yields a negative goodness of fit, which is clearly invalid and impossible (Schalken & Chantler, 2018 ▸; Trevorah *et al.*, 2020 ▸). Ergo, some might argue that the data do not permit a fit of even a single independent free parameter. If the *R* range was simultaneously fit over, for example, 1 Å < *R* < 4 Å (a fraught process), then the perception would be that only 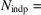 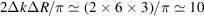 parameters could be defined, with ‘ 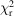 ’, by that definition of thousands, quite untenable in valid statistics. Complex arguments in the XAFS literature have suggested that the correct *N*_indp_ may be larger or smaller by one or two, but this, in any case, yields a negative goodness-of-fit metric and remains nonsensical. The solution, of course, is to use the actual number of independent data points, *N*_idp_, which in these individual datasets is of order 200 or so, and is of course better in other datasets or if simultaneously fitting multiple datasets. Let us reiterate that it is perfectly possible to define 15 near-independent parameters to very high accuracy if data uncertainties are measured individually and used in a valid goodness-of-fit metric.

A second concern is that this structure is very complex and has many local atoms, many bond lengths and angles, and hence many more parameters than the 15 fitted. Conventional XAFS analysis would fit the few nearest-neighbour values and assume that multiple legged paths and distant scattering shells were insignificant – though in fact both are significant, especially in the lower *k* range. On this detail, we follow the modelling of Streltsov *et al.* (2008 ▸), which models the quantum system for XAFS in a very similar manner to that used in crystallography and X-ray diffraction – that is, with known *a priori* information, constraints and restraints from biological molecular models. This is how it has become possible to gain insight into so many critical parameters.

The goodness-of-fit measure was weighted least squares including the estimated uncertainties of the experimental data, as contrasted with past conventional XAFS analysis on most systems, which uses a *post facto* constant uncertainty estimate and unweighted fit. The fitting was performed with *k* ^0^ weights of χ(*k*) data in *k* space with 3.0 Å^−1^ ≤ *k* ≤ 12.0/10.0/11.0 Å^−1^ for Cu^II^:Aβ_4–16/12/8_, respectively. Note that, unlike most XAFS analysis, because we propagate uncertainty to χ, fits in *k* ^*n*^ are or should be identical irrespective of *n* = 0, 1, 2, 3, with identical output and uncertainties. That is, the scaling of data and the scaling of uncertainty must be and are isomorphic. The *eFEFFit* script for the model is given in the supporting information.

**Figure 3:**
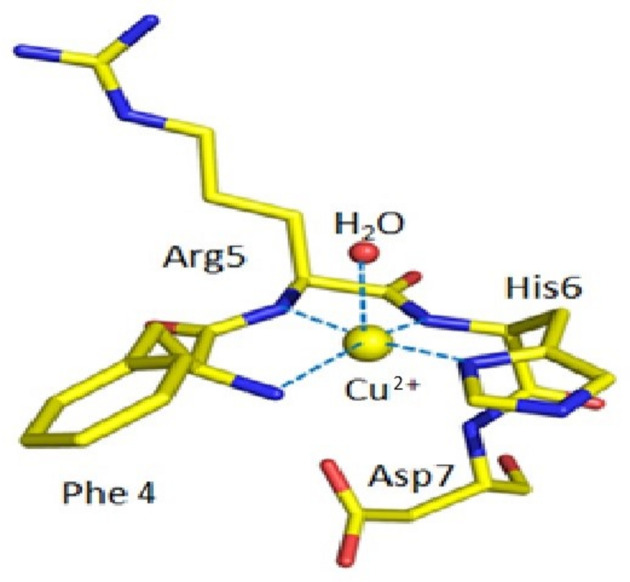
The best-fit model for Cu^II^ binding proves the dominance of an ATCUN-binding site (from *F* tests), with a water molecule in the site in N-truncated Aβ_4–*y*_ peptides. This suggests that the ATCUN site chelates Cu^II^ ions into its ring. It also clearly fails to suggest any pleomorphism.

A three-dimensional Cu^II^:Aβ_4–*y*_ structural model based on the reported density functional theory (DFT) for the Cu^II^:Aβ_4–16_ structure (Mital *et al.*, 2015 ▸) was initially constructed for fitting the EXAFS. The first shell of Cu^II^ coordination in this ATCUN-binding site for Cu^II^:Aβ_4–*y*_ peptides has an arrangement of four nitrogen ligands in equatorial positions, including the phenylalanine amino group N(Phe4), two deprotonated amides from the first two peptide bonds – N(Arg5) and N(His6), and an N atom of the imidazole side chain of the histidine residue ND1(His6). Here, we introduce a new model including an additional fifth coordination oxygen along the apical Jahn–Teller distortion axis, similar to the structure of the Cu^II^:DAHK peptide complex determined by single-crystal X-ray diffraction (Hureau *et al.*, 2011 ▸). This new model gives the best fit. Fig. 3 ▸ shows the Cu^II^ with the water molecule in the ATCUN-binding site for Cu^II^:Aβ_4–*y*_ peptides.

Variance and noise between datasets are primarily due to low-temperature noise and ice defects in regions of individual spectra and pixels. The consistency of the spectra between all of the Cu^II^:Aβ_4–8/12/16_ datasets suggests that the Cu^II^ coordination geometry does not change noticeably for the three peptides and that therefore it must be dominated by the Cu^II^:Aβ_4–8_ peptide. This in turn suggests that the Cu^II^-binding geometry of Cu^II^:Aβ_4–8/12/16_ is dominated by the high-affinity ATCUN site, which appears unaffected by residues beyond *y* = 8. The detailed fits confirm this hypothesis (Table 1 ▸).

Remarkable similarity of the parameters of the ATCUN fitting model across peptides confirms the non-involvement of Tyr10 or Glu11 in Cu^II^ binding, again in a stronger proof than that of the plotted-fit consistency.

Repeated measurements do not show the loss of amplitude and blurring of spectral features expected from radiation damage. If radiation damage permitted alternate binding (trigonal binding has been reported), we would expect to see developing pleomorphism and blurring of features, but these are not seen in the data.


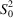
 is theoretically expected to represent the many-body relaxation of all the electrons in the absorbing atom to the hole in the core level, and should therefore theoretically be less than or equal to unity within uncertainty. This amplitude-reduction factor ( 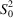 ) has been claimed to be about 0.9 for a copper compound (Poiarkova & Rehr, 1999 ▸). Similarly, it has been stated that 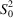 should be 0.9 ± 0.1 for a good fit of a sample (Levina *et al.*, 2005 ▸; Ellis & Freeman, 1995 ▸). We obtain 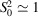 within 1–3 standard deviations for all peptides at low temperature. 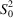 was a free parameter refined in the fits. In this analysis, the result is fully consistent with conventional literature expectations. Predictions of 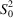 are highly model- and energy-calibration dependent, and do not currently reflect advanced theoretical expectations.

Experimentally, 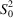 is highly correlated with the coordination number of nearest neighbours (*N*). This then confirms very well the value of the coordination number *N*, which is 100% correlated with any incorrect value of 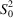 . We can therefore say clearly that *N* is accurate to better than 10%. Whilst *N*_*i*_ is an integer for each shell and is not a free parameter for a given model, the strong evidence for the apical water site confirms the very strong evidence for the given coordination numbers.

The energy (calibration) offset relative to the monochromator setting δ*E* is less than 10 eV, and robust around 3.5–4.3 eV. If the energy axis was significantly in error (*e.g.* too high), this correlates with an effective apparent decrease in 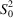 or *N*, and with an error in the fitted shell or path radii *r*_*j*_.

The shell or path radii *r*_*i*_ have uncertainties, especially for the inner shell, remarkably below 1 pm for individual scans, even though the data are relatively short range and noisy. These values are largely consistent with different scans and peptides within uncertainty. This is a direct consequence of reasonable and propagated uncertainties.

We report both χ^2^, relevant for hypothesis and model testing and *F* tests, and 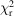 , which is the usual marker for goodness of fit. None of these are scaled or rescaled. A good statistician might be concerned that 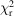 is a bit less than unity, indeed varying for an individual fit from 0.69 to 0.23. In fact, these values support our uncertainty estimates to within a factor of 2. If the uncertainty estimate is the dominant issue, then the uncertainties in the table of parameters would be reduced by 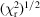 , *i.e.* by 20% or 50% in the most significant case, and hence would be even smaller than stated. We provide the model-dependent δχ^2^ for discussion of model and hypothesis testing.

### 4.3. Multiple data fitting in *eFEFFIT* XAFS for consistent datasets

**Figure 4:**
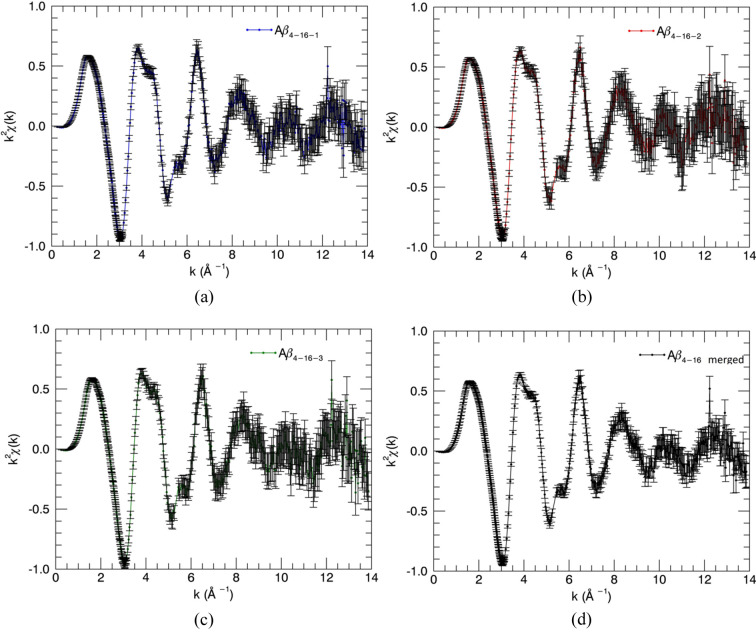
χ(*k*) oscillations of X-ray fluorescence spectra for Cu^II^:A 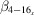 (*x* = scan 1, 2 or 3) and their merged, weighted mean, Cu^II^:Aβ_4–16_(black) χ(*k*) spectrum with uncertainties.

Fig. 4 ▸ shows the estimated uncertainties propagated from raw data to χ versus *k* for individual scans and then for a merged dataset. We can fit the merged datasets, or we can simultaneously fit multiple datasets with the same model. Streltsov *et al.* (2018 ▸) fitted unweighted merged datasets for all peptides. Here, we use weighted simultaneous fits of multiple datasets, to prove both the consistency with the individual fits and the consistency between the peptide fragments.

The *eFEFFit* package previously used only one EXAFS scan for structural refinements. We developed the *feffit2.f* and *iff-feffit2.f* code, subroutines in *eFEFFit*, so that multiple EXAFS scans can be used to refine structures. Three Cu^II^:Aβ_4–8/16_ EXAFS scans and repeated Cu^II^:Aβ_4–8/12/16_ scans were simultaneously fitted to the Cu^II^-binding model after confirming the consistency from *eFEFFit* individual refinements. The weighted average of repeated scans was used for each peptide. Table 2 ▸ provides the fitted results. The first and second columns give the multiple-scan fit results of Cu^II^:Aβ_4–8/16_ while the third column provides the multiple-scan fit results of Cu^II^:Aβ_4–8/12/16_.

**Figure 5:**
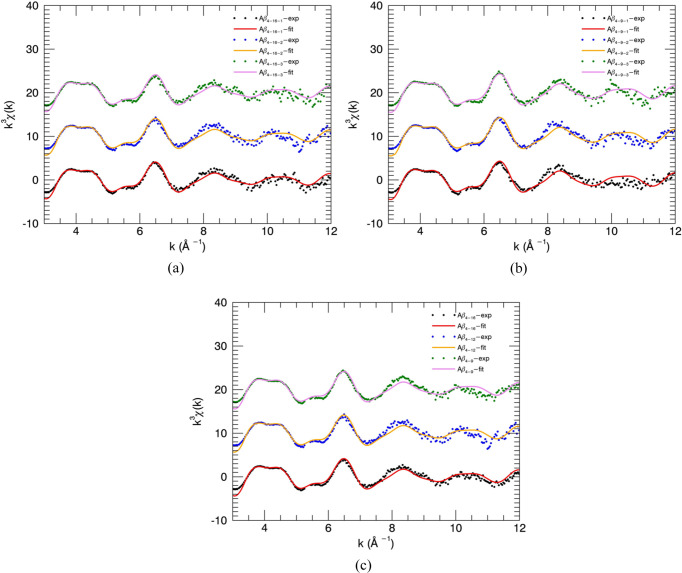
*k* ^3^χ(*k*) plots of multiple-scan EXAFS analysis from *eFEFFIT* in *k* space. (*a*) Three repeated measurements of Cu^II^:Aβ_4–16_. (*b*) Three repeated measurements of Cu^II^:Aβ_4–8_. (*c*) Averaged scans of Cu^II^:Aβ_4–8/12/16_.

It is encouraging to compare multiple-dataset refined parameters with individual refined parameters to control the quality of new development in *eFEFFIT*. The multiple-dataset refined values (Table 2 ▸) are consistent with the individual refined EXAFS measurements (Table 1 ▸). The fits of the model to multiple EXAFS scans in *k* space (Fig. 5 ▸) remain in good agreement with the experimental measurements. The multiple data refinement involves more experimental data points, enabling an increase in individual parameters for a better fit. Therefore, uncertainties are generally reduced, and accuracy is increased where the datasets are consistent. In particular, the final pooled 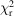 is unity, confirming strongly the estimated uncertainties and the propagation through the process of analysis, together with the use of valid goodness-of-fit metrics.

### 4.4. Cu^II^:Aβ discussion of data and literature concerns

The earliest XAS studies at room temperature suggest a *tris*-His site with tyrosine and perhaps oxygen (Stellato *et al.*, 2006 ▸; Minicozzi *et al.*, 2008 ▸; Morante, 2008 ▸) for Cu:Aβ_1–16_, and observe that deleting the first peptides suggests *bis*-His with tyrosine, N-terminal and oxygen for Cu:Aβ_5–23_. These reported estimates on path lengths have uncertainties of ± 0.01 Å, σ^2^ ≃ 0.002 (1) Å^2^. The energy offset was relatively large, δ*E*_0_ = 11–14 eV.

Shearer & Szalai (2008 ▸) and Hureau *et al.* (2009 ▸) investigated Cu:Aβ_1–16_ at room temperature and concluded a *bis*-His Asp1 amine N backbone carbonyl square-planar coordination, with *bis*-His and 2 N/O distorted square-planar geometry. Shearer *et al.* (2010 ▸) investigated Cu:Aβ_1–42_ at room temperature and concluded a *tris*-His N with one additional unidentified N/O in a square-planar geometry. None of these suggested pleomorphism.

Streltsov *et al.* (2008 ▸) suggested *tris*-His binding and two Asp1/Glu11 carboxylate oxygen binding with one axial water in a distorted octahedral coordination for Cu:Aβ_1–16_ at low temperature (20 K). An impressive 10–13 repeat scans of each species were performed, taking 40 min each. Radiative damage was observed. Radii were accurate to ± 0.007–0.01 Å, and σ^2^ of the first shell was 0.0020 (5) Å^2^. They provided strong evidence that Tyr10 was not involved in these bindings. All these scans investigated Cu:Aβ_1–16_ and not the common truncated Cu:Aβ_4–*y*_.

Streltsov *et al.* (2018 ▸) directly investigated Cu:Aβ_4–*y*_ at low temperature (10 K). Their δ*E*_0_ was perhaps relatively large (6.33 eV) and their radii had large uncertainty (0.14 Å, 0.04 Å), with a σ^2^ of 0.0035 (4) Å^2^, but some of the raw data were in common with the current work. Streltsov *et al.* (2018 ▸) suggested a tetragonal pyramid geometry for Cu^II^ binding in N-truncated Aβ_4–*y*_ using EXAFS analysis, though based on an estimation of uncertainty as a constant (ε) in *k* ^*x*^χ space. Conversely, our study finds a δ*E*_0_ of 3.6 eV; radii with uncertainty 0.006 Å, 0.01 Å; and a σ^2^ of 0.0089 (4) Å^2^. Distortions in 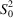 and Δ*E*_0_ can be seen in the earlier results, which are addressed in the current analysis. The ordering of the nearest-neighbour bonds changes, for the two closest contacts, although some of these were indistinguishable within uncertainty in the more standard analysis. For the same radius, element and oxidation state, XAS analysis does not distinguish between which nitrogen is the closer contact but it can identify their separation. This current work shows the apical water to be more tightly bound, though both studies show a high variance (σ^2^) on that bond distance, even at low temperature, due to the relatively weak potential binding and probably structural variation. That study could model only 10, 8 or 6 ‘independent’ parameters, whereas the current advanced analysis can measure 15 or so, while still improving the accuracy of each independent bond length, often by a factor of 14, by propagating the uncertainties with a valid goodness-of-fit criterion. Using and propagating estimated uncertainties dramatically aids fitting experimental data.

### 4.5. Cu^II^:Aβ questions of pleomorphism

Pleomorphism is known to exist in Aβ fragments, Aβ_1–42_ and *e.g.* Cu^II^:Aβ_1–42_. Which is to say that the different fragments, including under different pH *etc*., fold or bind in more than one way, and hence one will see a mixture of different molecular shapes, contacts and bindings to the central Cu atom. This will then blur X-ray diffraction maps and XAFS modelling, or perhaps reveal multiple components from a principal components analysis (PCA) and related techniques. Simple isomerism of the molecule, by contrast, may be unobservable and indistinguishable by XAFS, since the local bond distances, bond angles and related structure may be identical. Similarly, structures that only begin to differ at the fourth coordination shell, or at angles at far distances, may also be indistinguishable. We and others have proven that third shells, and three-leg and four-leg paths are explicitly observable with high-quality data. Our current study primarily fits the inner two shells, so is quite plausible. The critical questions here are whether Cu^II^:Aβ_4–8_ is one structure (monomorphic), even in solution, at least in near-physiological pH, and similarly Cu^II^:Aβ_4–12_ and Cu^II^:Aβ_4–16_; whether the binding site and structure are consistent and self-similar within the region of the first three shells, representing the same binding structure; and how to measure this in the data and analysis.

Summers *et al.* (2019 ▸) investigated Cu^II^:Aβ_1–42_ at low temperature (10 K) and different pH values (especially 6.1, 7.4 and 9.0), and concluded that the coordination number *N* of nearest neighbours varies from ∼4 at low pH to ∼5 at high pH. They confirmed that the local binding is sensitive to photoreduction, and suggested that only one His bond is present below a pH of 7.4 and that perhaps two are bound above that pH. They also made a careful summary of conclusions from experiments on various Cu^II^:Aβ peptide fragments, using a variety of experimental techniques. This literature, as discussed in Section 1, predicts any of: square planar, distorted square planar, tetragonal, square pyramidal and distorted octahedral nearest-neighbour geometries. However, we expect different fragments to have different binding, and we have proven this above.

Summers *et al.* (2019 ▸) raised four major concerns about investigating Cu: or Zn:Aβ fragments, and specifically Aβ_1–16_. One concern was whether the binding is of the monomer, or if aggregation had occurred and the target structure was an oligomer. Some authors have claimed that Aβ_1–16_ does not aggregate. With XAFS analysis, the local structure can be determined irrespective of this concern. Shearer *et al.* (2010 ▸) considered explicitly oligomeric Cu:Aβ_1–42_. It remains unclear what impact this might have had on the structure.

Summers *et al.* (2019 ▸) concluded that pleomorphism exists across pH and buffer choice, and especially from pH 6.1 through to 9.0 and at near-physiological 7.4. They also claim that the structure at near-physiological pH is not a mixture or PCA combination of reference structures at low and high pH. Pleomorphism has been suggested for Cu:Aβ_1–16_ in studies using other techniques from 2009 through to 2014. Interestingly, Summers *et al.* (2019 ▸) also collected high-energy resolution fluorescence detection XAS (HERFD-XAS) data, which should have higher resolution and may require fitting with different theory. Indeed, their HERFD-XAS structures show higher resolution, and also show different structure from their XAS scans. They conclude that the structure at near-physiological pH (7.4) may be a different dominant coordination than those from low and high pH, but is probably multiple pleomorphic. Their second concern would then be interpreted as the following: most studies, especially using XAFS, assume a single binding structure without pleomorphism, whereas they look for an ‘averaged structure’ but by fitting a single structure. This is isomorphic to fitting a single structure.

Summers *et al.* (2019 ▸) concluded that, given an assumption of pleomorphism, solving for multiple structural components using standard XAFS is difficult or infeasible; and that structural information will be heavily limited. Furthermore, solving for a single component will either be invalid or yield high 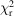 or a distorted structure, from the heterogeneity of the local environment. We find no such evidence, either in our data or theirs (see especially the discussion on Cu^I^ below). They also appear to have presented no experimental evidence in favour of this pleomorphism or the multiple-component composition.

### 4.6. Cu^II^:Aβ questions of data information content for XAFS analysis, and radiation damage

A third concern raised by Summers *et al.* (2019 ▸) is the ‘reluctance to dismiss Tyr10 in coordination’, though, indeed, for Cu:Aβ_1–16_, Streltsov *et al.* (2008 ▸) had explicitly investigated this with structural tests. Similarly, here, we also investigate this for Cu:Aβ_4–*y*_, and the results are conclusive.

Summers *et al.* (2019 ▸) stated that EXAFS analysis has very heavy (stringent) limits on the determination of backscattered distances, including the number of scattering shells, and the ability to identify or quantify nearby radii, due to low-resolution *R*-space data, in turn caused by limited or even broad *k* data. They claimed that it is infeasible to determine δ*r*_*i*_ < 0.12 Å for EXAFS data only fitting or collected from *k* ≃ 0 up to *k* = 13 Å^−1^, considering a Nyquist-like prescription commonly used in the XAFS community and attributed incorrectly to Nyquist. Similarly, they claimed that results reported in earlier Aβ fragment studies by EXAFS analysis of related peptide samples (Stellato *et al.*, 2006 ▸; Minicozzi *et al.*, 2008 ▸; Streltsov *et al.*, 2008 ▸) were invalid due to these resolution limitations, that is, minimum separations of radii of nearby shells should have been 0.16 Å and 0.13 Å, respectively. These papers conclude that separations of 0.020 (8) Å are meaningful, for example, for Cu^II^:Aβ_1–16_ and Cu^II^:Aβ_1–42_ (Streltsov *et al.*, 2008 ▸). Summers *et al.* (2019 ▸) claimed that EXAFS fitting of multiple backscatterers at similar distances separated by less than 0.12 Å to 0.16 Å would return an artificially inflated coordination number due to false (phase) cancellation of the respective photoelectron waves. This limitation issue is commonly cited but not discussed in EXAFS analysis with Fourier-transform data. We do not directly address the earlier Aβ fragment experiments on this topic because uncertainties were not defined or propagated to the final fits, so the uncertainties might be underestimated. We agree that components and parameters, including shell radii, which are highly correlated (*i.e.* very similar radii), are therefore challenging or sometimes impossible to define.

However, our experiments and this article defined and propagated uncertainties, so that the parameter uncertainties are at least robust. In particular, maths and information content has never required a full separation of a wave component to define it; rather, it requires sufficient data accuracy and spacing to separate components, which are of course always overlapping. Notably, Summers *et al.* (2019 ▸) give reference examples where they have reported features at smaller separations than the false Nyquist assumption.

Closely spaced shell radii can be distinguished with high-accuracy experimental data and well defined experimental uncertainties and noise. If the uncertainties are too large or the parameters are not independent then the resolution of the shells is weaker (Trevorah *et al.*, 2020 ▸). Our fitted determined shell radii are given to high accuracy from ±0.04 down to ±0.0001 Å. In particular, we determine meaningful shell separations for Cu^II^:Aβ_4–*y*_ down to 0.018 (6) Å, far below a naive 0.16 Å limit. Our results prove that *N* is not skewed. An integer error of *N* would represent a 20% error of backscatterer cancellation, which is not observed. We compute 10% or greater contributions of multiple scatterings in the fits. This supports a number of the previous experimental findings, including Streltsov *et al.* (2018 ▸), on this point, though more research will be valuable.

Summers *et al.* (2019 ▸) claimed that the range 0.0015 Å^2^ ≤ σ^2^ ≤ 0.0080 Å^2^ is the physically plausible range for the isotropic thermal parameter, that values above this range are unreasonable as the structure from each wave would be hard to extract from software, and that values below this range are unreasonable because there must always be some inherent vibration. Of course, this statement is temperature dependent. It is also pleomorphism dependent (see below). They comment that many of the previous XAFS fits have σ^2^ ≃ 0.008–0.009 Å^2^, and that therefore the associated fit components are highly dampened (broadened) and therefore probably unrealistic. We agree that extreme values of σ^2^ are a useful marker of possible problems with the analysis or the quality of the data.

Conversely, our EXAFS measurements with propagated uncertainties accurately fitted including peaks at higher *k* (Fig. 2 ▸) and refined consistent and physical innermost-shell σ^2^ values in the range of 0.001 Å^2^ ≤ σ^2^ ≤ 0.0089 Å^2^ within the uncertainties. Trevorah *et al.* (2020 ▸) conducted more critical analysis to observe significant features and peaks in spectra with reported higher σ^2^ and proved the possibility of a low value of σ^2^ within the refined errors. Our best fitted values are comparable within the given uncertainty with the XAS refined values for Cu^II^Aβ_4–*y*_ (Streltsov *et al.*, 2018 ▸), Cu^II^Aβ_1–16/42_ (Streltsov *et al.*, 2008 ▸) and the Cu^II^ imidazole system (Binsted *et al.*, 1992 ▸), and with the values obtained for the similar systems (Poiarkova & Rehr, 1999 ▸; Dimakis & Bunker, 2002 ▸).

Summers *et al.* (2019 ▸) concluded that Cu^II^ coordination in monomeric Aβ_1–42_ resulted in multiple conformations in a range of pH solutions (*i.e.* it is intrinsically pleomorphic and pH dependent). The latter is certainly true and shown by their data. However, they provide no evidence for pleomorphism from their data or fits and do not attempt to fit multiple components. If the pleomorphism was strong, and shell radii from different geometries were overlapping, then we would expect a high σ^2^ from the blurring of shells. Conversely, we find strong evidence for EXAFS data fitting for a single species under our experimental conditions. Goodness-of-fits χ^2^ and 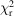 reflect the validity of the fitted model, where

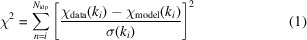

and 

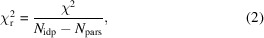

where σ(*k*_*i*_) is the associated propagated uncertainty in *k* space. More concerningly, Summers *et al.* (2019 ▸) used a fit error function defined as 

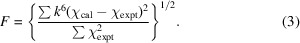

This error function forces the data to highly weight the high-*k* data points, where the amplitude and signature are dominated by the single-closest-shell radius, so that the others are indeed poorly defined. And it does not weight the range of the data equally. Thirdly, σ(*k*_*i*_) is not measured and is set to a somewhat arbitrary constant for all *k*, even after scaling by *k* ^3^. More importantly, this is not an appropriate goodness-of-fit function, whether for least-squares analysis or for Bayesian analysis. It is unfortunate that this error function was used, and it would be interesting and instructive to repeat their experiment with defined uncertainty and propagation, and to use hypothesis testing for any pleomorphism that may or may not be present.

There was a large and excellent discussion by Summers *et al.* (2019 ▸) of the prevalence of radiation damage, including in their results. We discuss this in detail in another paper (Ekanayake *et al.*, 2024 ▸) and prove that we have no observable radiation damage.

### 4.7. Discussion of Cu^II^ results in N-truncated Aβ_4–8/12/16_ peptides

There are different types of Cu^II^ coordination proposed or believed in Aβ_*x*–*y*_ peptides. We have demonstrated that ATCUN-type binding coordination is dominant for Cu^II^ binding in Aβ_4–*y*_ peptides. Huang *et al.* (1999 ▸), Curtain *et al.* (2001 ▸) and Drew *et al.* (2009*a* ▸) proposed three N and one O for Cu^II^ coordination, while Streltsov *et al.* (2008 ▸) introduced three N, three O and axial water. Faller & Hureau (2009 ▸) discussed two N and one O for Cu^II^ coordination in Aβ peptides. Our model suggests four N and a water molecule in an axial direction for Cu^II^ coordination in Cu^II^ binding in Aβ_4–*y*_ peptides, strongly arguing against these earlier models. However, our suggestions confirm the binding ligands suggested by Streltsov *et al.* (2018 ▸) for Cu^II^:Aβ_4–12/16_ with more reliable answers obtained from the advanced EXAFS analysis.

Structural refinements without the water molecule confirmed improvement of the suggested Cu^II^-binding model. The significance is supported with the statistical *F* test by generating calculated *F*_2, 31_ = 4.33, which is greater than the tabulated criterion of *F*_2, 31, 0.05_ = 3.30 at the common significance level of α = 5%. The refined distances of the Cu^II^ ion for the equatorial nitrogen atoms and apical water were obtained ranging from 1.831 (8) to 2.05 (1) and 2.13 (2) Å, respectively. The findings of the current study are consistent within the uncertainties with those of reported values for the Cu^II^:DAHK complex in crystallography (Hureau *et al.*, 2011 ▸). Similarly, Camerman *et al.* (1976 ▸) illustrated the Cu^II^ chelating of ATCUN. They discussed a square-planar-binding arrangement with Cu–N distances and a weak O interaction with Cu–O. These results are also consistent with ours. These comparisons suggest the possibility of Cu^II^ ATCUN binding with an axial water in tetragonal pyramid geometry for N-truncated Aβ peptides.

These results corroborate those observed in earlier studies by Mital *et al.* (2015 ▸), as they discussed the Cu^II^ binding with the ATCUN motif. However, Mital *et al.* (2015 ▸) stated that three N atoms and one O atom are involved in the binding. Similarly, Huang *et al.* (1999 ▸) and Curtain *et al.* (2001 ▸) discussed the Cu^II^ binding with three N atoms and an O atom in peptides. Our studies contradict these earlier findings, proving a new Cu^II^ binding with four N and one axial water in the fifth position. The controversial discussion on the involvement of tyrosine in Cu^II^ binding is addressed from our findings. Stellato *et al.* (2006 ▸), Mital *et al.* (2015 ▸) and Wiloch *et al.* (2016 ▸) have argued that Tyr10 has been associated with the Cu binding; however, our findings have evidently disproved that argument, certainly for Aβ_4–*y*_, justifying Karr *et al.* (2005 ▸) and Streltsov *et al.* (2018 ▸) on this point.

Streltsov *et al.* (2018 ▸) proved that a significant number of independent parameters can be fitted in XAFS spectra, even when the number of data points and the *k* range are limited, and that complex bio-systems can be modelled and investigated using *a priori* peptide and biological and chemical constraints. Herein, in this article and in this section, we have investigated a series of hypotheses for low-*T* Cu^II^ results in N-truncated Aβ peptides. These hypotheses are summarized in the following paragraph (‘confirmed’ means ‘proven’).

(1) The bonding (model spectra and structure in quantitative detail) of sequential Cu^II^:Aβ_4–8_ datasets is self-similar, and separately the bonding of sequential Cu^II^:Aβ_4–16_ datasets is self-similar – confirmed, showing no broadening due to radiation damage (which normally lowers the coordination number and hence induces pleomorphism). (2) The thermal parameters for bond lengths in all (each) peptide fragments studied are consistent with a single molecular structure – confirmed, showing no observable (measurable) pleomorphism. (3) The structures refined in all cases for each peptide fragment scan show no observable multiple component or multiple species – confirmed, no pleomorphism. (4) The detailed structural fits of each scan and of all seven scans in a multiple fitting with uncertainty weights are self-similar for Cu^II^:Aβ_4–8/12/16_ datasets – confirmed, proving that the bonding in these three fragments is self-similar at least out to the third bonding shell, noting that there are significant data contributions from at least the fourth coordination shell and from four-leg paths in the data and in the fit; this also proves quantitatively the absence of Tyr10 and Glu11 binding in these structures. Clearly these fragments cannot bind histidines (His13 or His14) either, yet the binding is strong. (5) The detailed consistency of all fits proves that the structure is bound in an ATCUN-binding site, as previously suggested – confirmed, though the ordering of bonding of the four nearest neighbours is different. (6) The binding site is five-coordinated with an apical oxygen (probably water), with more significant variability, even at 10 K – confirmed, proving that the water can oscillate in the weak apical potential; incidentally, a second apical oxygen for distorted octahedral geometry was also tested, but is not supported by the data. (7) The uncertainty estimates measured as a function of *k* are as measured – confirmed; they appear initially to be accurate within no worse than 50% uncertainty on the uncertainties, on the basis of the structural significance and the fitting 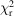 , but the detailed analysis and 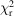 suggest an accuracy of estimates better than 20%, or even with the multiple weighted fit of seven scans to be within 2%. (8) Hence the choice and number of model parameters fitted are close to a limit on the basis of the quality of these particular datasets – confirmed. (9) The site for N-truncated Cu^II^:Aβ_4–*y*_ peptide fragments is a completely different site and bonding from that of Cu^II^:Aβ_1–*y*_ peptide fragments – confirmed. (10) The coordination number is 5 to within better than 10% – confirmed. (11) The detailed data analysis with defined (measured) uncertainties as a function of *k* avoids significant distortions of *e.g.* Fig. 2 ▸ and Tables 1 ▸ and 3 ▸ compared with earlier work, even with data of limited statistical quality – confirmed, and these plots in *R* space are from fits solely in *k* space with no *k* ^*n*^ weighting, precisely because the *k* weighting does not apply if the uncertainties are measured or quantified. (12) It is completely possible to have detailed and accurate insight into fragile biological solutions and active sites with limited but well defined statistics down to 15 parameters, bond-length accuracies of 0.01 Å or 0.3%, shell separations of 0.018 ± 0.006 Å and a 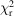 of unity (1) – confirmed. (13) This can then be used for statistical hypothesis testing of a tenth bonding parameter of a weak or blurred signature with careful *F* testing – confirmed. (14) To achieve this, it is recommended as necessary to use valid statistical measures of inference, even with modern caveats.

In the next section, we present a series of similar hypotheses for photoreduced species at room temperature in the electrochemical cell. The photoreduction can in principle yield Cu^I^ in some binding sites, or Cu^0^ in metal form. We particularly additionally investigate the following questions. Is the photoreduced structure stable? Is it monomorphic? Is it the same binding or the same peptides? Is it four or five coordinate? Are the conclusions statistically valid?

## 5. Cu reduction and Cu^I^-binding ligands in N-truncated Aβ_4–8/12/16_ peptides

### 5.1. Identification of Cu^I^-binding ligands from XANES

Electrochemistry drives the potential of the species to reduction or oxidation. Under reduction, any sample solution could be a mixture of Cu^II^ and Cu^I^ if the reduction has occurred during the electrolysis and if it is partial. A qualitative XANES analysis is performed to identify the Cu^II^ to Cu^I^ reduction and the involvement of residues in the peptide with the reduction process.

In this experiment, the reduction of copper ions in the peptide sample solution failed to give a current response that was distinguishable from the background signal, due to slow electron-transfer kinetics (Streltsov *et al.*, 2018 ▸), which is consistent with the observations of Mital *et al.* (2015 ▸). However, perhaps surprisingly, the reduction of the sample can be recognized by significant changes in the XANES spectra.

**Figure 6:**
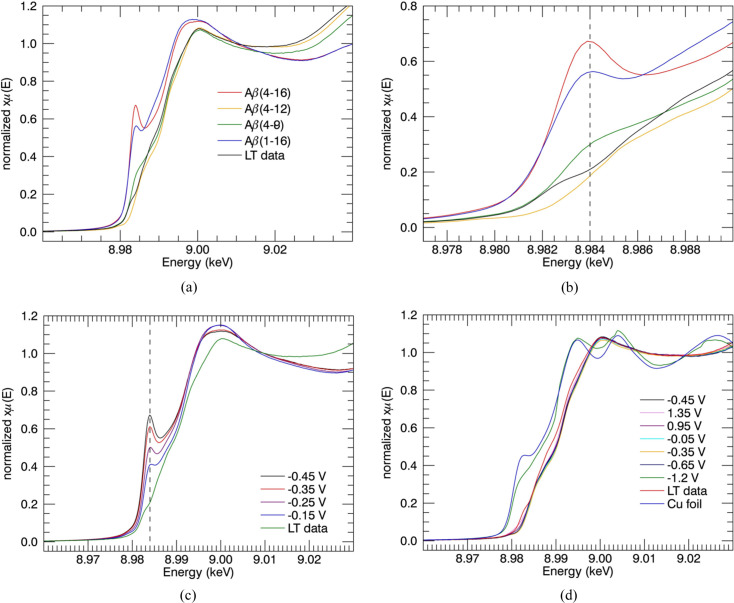
(*a*) Room-temperature XANES-EC spectra for Cu:Aβ at a reduction potential of −0.45 V. Reducing potentials for Cu^II^:Aβ_1–*y*_ peptide complexes in the literature are in the range of 0.28–0.34 V (Guilloreau *et al.*, 2007 ▸; Jiang *et al.*, 2007 ▸). Changes in the potentials depend upon the time allowed for the equilibrium of species. (*b*) Enlarged XANES spectra showing the Cu^I^ characteristic peak at 8984 eV corresponding to the 1*s*–4*p* transition. (*c*) Room-temperature XANES-EC spectra for Cu:Aβ_4–16_ at a series of reducing potentials from −0.15 to −0.45 V. (*d*) Room-temperature XANES-EC spectra for Cu:Aβ_4–12_ at different reducing potentials from −0.05 to −1.20 V. Plots are compared with the low-temperature (LT) and Cu-foil XANES spectra.

XANES provides reliable evidence for the reduction of Cu^II^ ion into Cu^I^ ion in the N-truncated Cu^II^:Aβ_4–16_ peptide. The characteristic features of Cu^I^ are obtained at potential −0.45 V versus normal hydrogen electrode. The XANES spectra of Cu^II^:Aβ_4–16/12/8_ peptide complexes at the reduction potential are plotted [Figs. 6 ▸(*a*) and 6 ▸(*b*)]. The XANES series obtained at the potentials of −0.15 V, −0.25 V, −0.35 V and −0.45 V for Cu^II^:Aβ_4–16_ peptide are given in Fig. 6 ▸(*c*). XANES-EC regions for Cu^II^:Aβ_4–12_ at potentials from 0.95 to −1.20 V are given in Fig. 6 ▸(*d*).

Low-temperature XANES is also plotted for comparison. The characteristic Cu^I^ peak is observed at 8984 eV. The moderate changes in the potential are insensitive to the Cu^I^ spectra, justifying the reduction of Cu^II^ at room temperature. Metal-based reduction of Cu^II^ ion to Cu^I^ in both the Cu^II^:Aβ_1–16_ and Cu^II^:Aβ_4–16_ complexes is observed [Figs. 6 ▸(*a*) and 6 ▸(*b*)].

**Figure 7:**
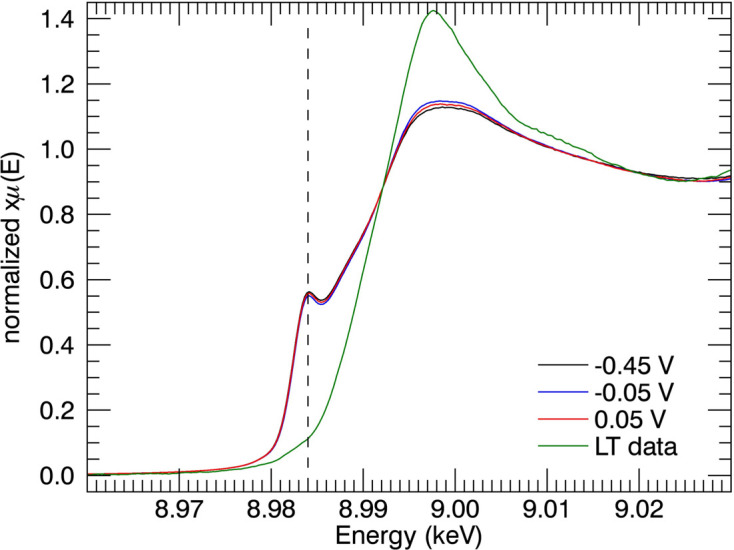
Room-temperature XANES-EC spectra for Cu:Aβ_1–16_ at reducing potentials from 0.05 to −0.45 V. The Cu^I^ characteristic peak is observed at 8984 eV corresponding to the 1*s*–4*p* transition. The pre-edge peak heights are slightly smaller than the reported spectra for reductions by ascorbate (Streltsov *et al.*, 2008 ▸).

The findings of the current study are consistent with Kau *et al.* (1987 ▸) – the XANES spectra demonstrate the characteristic peak at about 8984 eV, associated with the 1*s*–4*p* transition across the range 8980–8985 eV. A similar Cu^I^ peak was reported in metal reduction of Cu:Aβ_1–16/42_ peptides (Streltsov & Varghese, 2008 ▸). The pre-edge peak heights of the current Cu^II^:Aβ_1–16_ spectra (Fig. 7 ▸) are slightly smaller than the reported spectra for Cu^II^:Aβ_1–16_ reductions by ascorbate (Streltsov & Varghese, 2008 ▸). Hureau *et al.* (2009 ▸) suggest that a greater intensity obtained for the Cu^I^ pre-edge feature was due to complete reduction of Cu^II^:Aβ. The Cu^I^-binding geometry and linearity of the coordination are also associated with the intensity of the pre-edge peak. The linearity of two-coordinated Cu^I^ geometry would dictate the properties and productivity of redox reactions (Himes *et al.*, 2007 ▸; Shearer & Szalai, 2008 ▸).

The XANES spectra of Cu^II^:Aβ_4–12/8_ peptide at the above-mentioned potentials have insignificant changes from the low-temperature spectrum, demonstrating no photoreduction of copper ion over a long period of time (*ca* 30 min). A further reduction of Cu^I^ to Cu^0^ was observed with stronger (forcing) reduction potentials. However, more careful investigations can explore the Cu^0^ reduction. These experimental results with different-length N-truncated Aβ peptides prove not only the non-engagement of the ATCUN Cu^II^-binding motif in copper-ion reduction but also the involvement of some suitable coordination for Cu^I^ to access. Requirement of more forcing conditions for the reduction of Cu^II^:Aβ_4–12/8_ was observed, suggesting that the reduction of Cu^II^ in the ATCUN site is feasible at moderate potential for peptide sequences including H13H14.

An XANES-EC series of different reducing potentials from 0.05 to −0.45 V were given in Fig. 6 ▸. Past reported reducing potentials for Cu^II^:Aβ_1–*y*_ peptide complexes including Cu^II^:Aβ_1–16_ were 0.28 to 0.34 V (Guilloreau *et al.*, 2007 ▸; Jiang *et al.*, 2007 ▸). Changes in the potentials depend upon the time spent towards equilibrium of reduced and oxidized species for XAS-EC experiments. Mital *et al.* (2015 ▸) did not observe conventional electrochemical measurements for Cu^II^:Aβ_4–16_. These XAS-EC results demonstrate the reversible reduction of Cu^II^:Aβ_4–16_. This finding corroborates the reduction of Cu^II^:Aβ_4–16_ with cysteine and glutathione (Santoro *et al.*, 2017 ▸). Assuming that the same thermodynamic equilibrium constants are applied for Cu^I^ binding in both Aβ_1–16_ and Aβ_4–16_, then the *ca* 3000 times stronger Cu^II^ binding of Aβ_4–16_ in the ATCUN motif could change the reducing potential of Cu^II^:Aβ_4–16_ by *ca* 0.21 V compared with the reducing potential of Cu^II^:Aβ_1–16_. There has been a hypothesized intermediate preorganization site in Aβ_4–16_, which might be structurally related to the low-affinity Cu^II^ ATCUN-binding site, resulting in a current reduction rate dependent on kinetics of the preorganization electron transfer (POET) mechanism (Streltsov *et al.*, 2018 ▸). The involvement of H13H14 residues is significant for Cu^II^:Aβ_4–16_, while the absence of H13H14 residues shows reduction for Cu^II^:Aβ_4–8/12_ only to Cu^0^ under more reducing potentials.

### 5.2. Structure of the Cu^I^-binding site in Aβ_4–16_ from room-temperature EXAFS

The pre-edge features of the XANES spectra suggest a three-coordinate geometry of the Cu^I^ binding into the N-truncated Aβ peptide. Therefore, a comparable DFT-optimized model for Cu^I^ with N_2_O coordination was used as the initial model for EXAFS-EC fitting. Two imidazole N atoms (ND1) in *trans* coordination arrangement and a carbonyl oxygen atom from His13 of backbone amide are bound to Cu^I^.

The refined model allowed individual fitting of nine structural parameters, out to a single scattering path length up to 5 Å, with chemical-bond restraints. The independent fitting parameters were: δ*E*_0_ – offset of the photoelectron energy threshold; overall scaling (amplitude reduction factor) 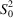 ; three independent thermal parameters for the axial water, for the nitrogen in His14 and for the oxygen in His13 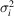 ; and four independent radial-adjustment distances. Four restraint functions are included to maintain reasonable parameters for 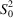 , 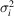 and two bond-length estimates. Multiple scattering contributions up to four legs with contributions of up to 10% were included in the refinement.

**Figure 8:**
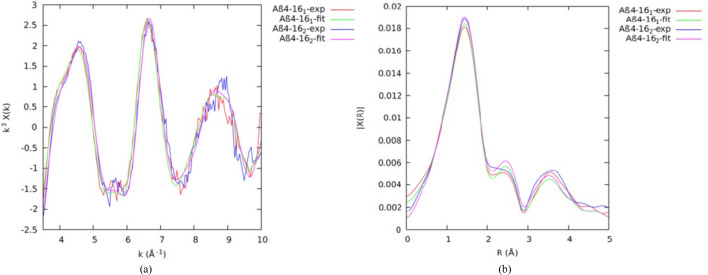
EXAFS analysis of Cu^I^:Aβ_4–16_ in (*a*) *k* space and (*b*) *R* space from *eFEFFIT*. Non-interpolated experimental data in *k* space were used for refinement, which preserves the information content from the data. An optimized interpolated cubic spline approach was utilized within the *mu2chi* package (Schalken & Chantler, 2018 ▸) to yield χ(*k*). The refined parameters are in Table 3 ▸.

**Figure 9:**
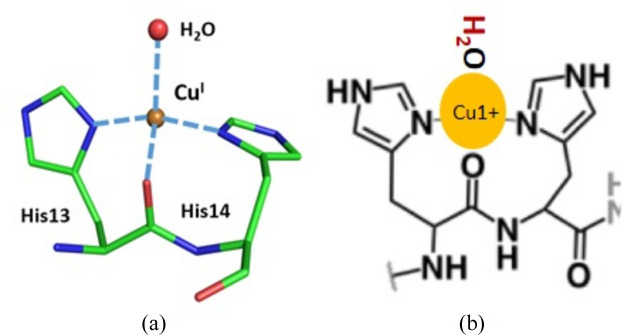
A schematic representation of (*a*) Cu^I^ binding into *bis*-His with an additional oxygen atom, probably from water, (Table 3 ▸) using *eFEFFIT* XAS data analysis for Cu^I^:Aβ_4–16_. (*b*) Chelation of a Cu^I^ ion into the *bis*-His site with a water oxygen atom in a quasi-tetrahedral geometry.

The fit was performed from *k* = 3.5 to 10 Å^−1^. The best fit obtained was with a quasi-tetrahedral environment, with the fourth coordination site occupied by the oxygen atom of a water molecule, O (water), Table 3 ▸ (Fig. 8 ▸). The significance of this improvement is supported by the statistical *F* test: *F*_2, 9_ = 6.53 is greater than the tabulated value of *F* distribution, *F*_2, 9, 0.05_ = 4.26, for the significance level of α = 5%. The consistency of the refined parameters of individual scans [Cu^I^:Aβ_4–16(1)_ and Cu^I^:Aβ_4–16(2)_] justifies the non-photoreduction of the measurements. The EXAFS fitting was improved by adding the fourth coordinating oxygen atom (water) (Fig. 9 ▸).

The four-coordinate geometry with quasi-tetrahedral N_2_O_2_ centre for Cu^I^ binding in Aβ_4–16_ is comparable with that of the N_2_OS centre for transmembrane Cu transporter protein (CTR1_1–14_) by XAS analysis (Pushie *et al.*, 2015 ▸). In our analysis, distances of the Cu^I^ ion to nitrogen atoms range from 1.803 (7) to 1.98 (1) Å. These findings are consistent within the returned errors with those for the CTR1_1–14_ (Pushie *et al.*, 2015 ▸; Streltsov *et al.*, 2018 ▸), and are more accurate with propagated uncertainties. Other ligands available in the Aβ_4–42_ sequence, for example sulfur (S) in glutathione (GSH) or methionine 35 (Met35), can substitute with the O (water) in the Cu^I^:Aβ_4–16_. It is widely held that Met35 in Aβ results in neurotoxic action. Misiti *et al.* (2010 ▸) discussed the association of Met35 with Aβ in generating ROS. Butterfield & Sultana (2011 ▸) discussed the understanding of Met35 of Aβ and its contribution of oxidative stress. There is a strong possibility that the oligomeric Cu^I^:Aβ species with Met35 can also induce neurotoxicity in the brain. The presence of soft donor atoms in peptides significantly affects reduction kinetics, especially in a POET mechanism where kinetics of equilibrium state happen during the reduction (Streltsov *et al.*, 2018 ▸). Hence, the structure of the oligomers and peptide conformations are important, as the propensity to generate ROS is different.

**Figure 10:**
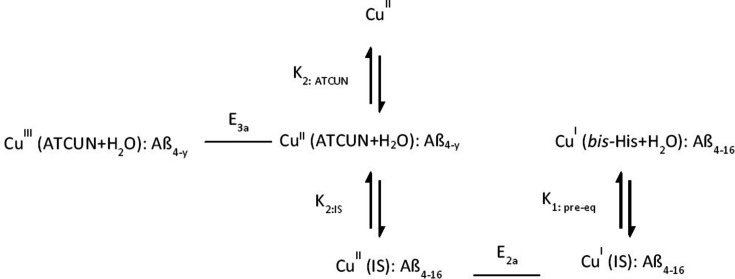
A modified latimer diagram that displays a summary of the redox chemistry for N-truncated Aβ peptides. This is based on the XAS-EC studies. The equilibrium arrows indicate the main component of the equilibrium. Cu^II^ from the ATCUN site ( 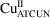 :Aβ_4–16_) to the *bis*-His site ( 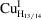 :Aβ_4–16_) could reasonably transfer via the H6H13/14 intermediate site (IS). Here, *E* is the redox potential and *K* is the equilibrium constant of the redox reaction.

The transfer of copper ion from the ATCUN site to the *bis*-His site with reduction could possibly proceed through the H6-H13/H14 intermediate site for Cu^II^:Aβ_4–16_, in the same way as in the Cu^II^:Aβ_1–16_ reduction process (Balland & Hureau, 2010 ▸). However, the XAS-EC results do not explain the details of the electron-transfer rate or of the intermediate-site geometry. The low-affinity intermediate Cu^II^-binding site is available at a ratio of 1.8/1 for Cu^II^/Aβ_4–16_ (Mital *et al.*, 2015 ▸). This intermediate binding site is possibly formed by the *bis*-His site with an additional residue including His6 (Fig. 10 ▸). This may be assisted by forming a four-coordinated Cu^I^-binding structure, which produced the best-fitted EXAFS results and an XANES pre-edge peak at ∼8984 eV.

Conventional voltammetry experiments for Cu^II^:Aβ_4–16_ returned no current response above the background (Mital *et al.*, 2015 ▸). Kinetics of Cu^II^:Aβ_4–16_ lower than Cu^II^:Aβ_1–16_ explain the more negative potential and the slower electron-transfer rate resulting in indetectable voltammetry responses for Cu^II^:Aβ_4–16_. The reduction rate of Cu^II^:ATCUN depends on the barrier to equilibrium to intermediate sites (Schwab *et al.*, 2016 ▸). Structural conformation and the length of the sequence are important for the reduction of an ion between two binding motifs separated by an amino acid for copper-bound protein complexes. This could be complicated with aggregation of metal-bound peptides in the form of oligomers or plaques.

These results indicate that the reduction of Cu^II^ in the ATCUN site of peptides is dependent on the availability of other accessible binding sites or ligands and dynamic stability connecting to copper binding into the sites.

XAS-EC data would expect a mixture of Cu^I^ and Cu^II^. Therefore, linear combination analysis (LCA) was applied to the room-temperature Cu^I^:Aβ_4–16_ data using the references of the low-temperature Cu^II^:Aβ_4–16_ fitted data and room-temperature Cu^I^:Aβ_4–16_ fitted data as the standards. The results show a Cu^I^/Cu^II^ ratio of 0.994/0.006 in the room-temperature mixture, within one standard error of unity (see Appendix *A* [App appa] for details).

## 6. Cu^II^ oxidation in N-truncated Aβ_4–8/12/16_ peptides

### 6.1. Qualitative identification of Cu^III^-binding ligands from XANES

**Figure 11:**
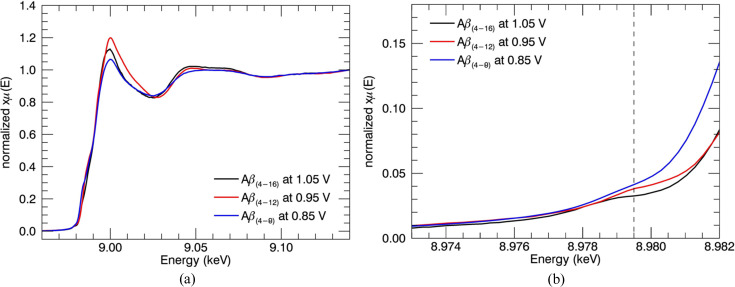
(*a*) XANES-EC spectra for Cu^III^:Aβ_4–12/16_ at oxidative potentials. (*b*) Enlarged XANES-EC spectra showing the pre-edge peak of Cu^III^ at 8979.5 eV, which corresponds to the 1*s*–3*d* electron transition in copper. Cu^III^:Aβ_4–8_ does not show any pre-edge feature at this energy, indicating no copper oxidation in the Aβ_4–8_ fragment.

We conducted an XAS-EC experiment to collect XANES spectra to investigate the occurrence of Cu^II^ to Cu^III^ oxidation in the solution. The changes related to an oxidation process were observed in the pre-edge region at an oxidative potential of 0.95–1.35 V during XAS-EC for Cu^II^:Aβ_4–12/16_. The oxidative process may be associated with the creation of Cu^III^ (Figs. 11 ▸ and 12 ▸). Similar types of irreversible oxidation peaks were reported for Cu^II^ complexes of the terminally blocked hexapeptide TESHHK from cyclic voltammetry data at pH 11.6 (Kaczmarek *et al.*, 2005 ▸). Generally, the presence of Cu^II^ shifts the oxidation peak to a more positive potential (Tsai & Weber, 1992 ▸).

**Figure 12:**
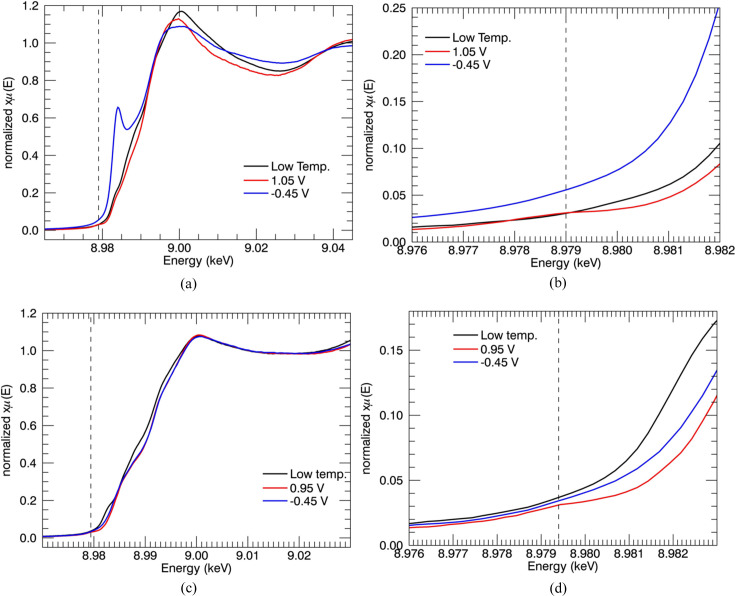
(*a*) XANES-EC measurements under the reducing potential of −0.45 V and the oxidative potential of 1.05 V for Cu:Aβ_4–16_ at room temperature. The low-temperature data are also given for comparison. (*b*) Enlarged XANES-EC spectra showing a slight peak at 8979 eV, corresponding to the 1*s*–3*d* transition, at the 1.05 V potential. Cu^II^ in the mixture slightly increases the oxidation potential. (*c*) XANES-EC measurements under the reducing potential of −0.05 V and the oxidative potential of 0.95 V for Cu:Aβ_4–12_ at room temperature. (*d*) Enlarged XANES-EC spectra showing a very slight peak at 8979.4 eV, corresponding to the 1*s*–3*d* transition, at the 0.95 V potential.

Our XANES spectra show a small intensity increase in the pre-edge region with a slight shift at 8979.5 eV for Cu^II^:Aβ_4–12/16_ [Figs. 12 ▸(*b*) and 12 ▸(*d*)]. This may illustrate the involvement of Cu^III^. This may also indicate any symmetry changes in the site. The small pre-edge peak at about 9879 eV could correspond with the 1*s*–3*d* electron transition in copper. A similar pre-edge peak was found at 8979.3 ± 0.3 eV for a 13-membered ring cyclic tetrapeptide *c*(Lys-DHis-βAla-His) (DK13)/Cu^III^ complex structure from XANES (Pratesi *et al.*, 2012 ▸). This supports a possible interpretation of oxidation from Cu^II^ to Cu^III^ in Cu:Aβ_4–*y*_ peptides.

No significant changes in XANES-EC were observed for Cu^II^:Aβ_4–8_ at 0.85 V suggesting that the oxidation could be tyrosine centred. Tsai & Weber (1992 ▸) investigated the influence of the Tyr10 residue in Cu^II^-peptide complexes. They illustrated the change of Cu oxidation reaction with the involvement of Tyr10, concluding that Tyr10 increases the oxidation and that the position of Tyr10 in the peptide is sensitive to the reaction.

### 6.2. Possible Cu^III^-binding site in Aβ_4–16/12_ peptides from room-temperature EXAFS

The presence of Cu^III^ oxidation states in Cu^III^:Aβ_4–16/12_ are suggested from XANES. A reliable 4N quasi-planar square structural geometry for Cu^III^ binding in a cyclic tetrapeptide *c*(Lys-DHis-βAla-His) (DK13)/Cu^III^ complex was reported from EXAFS and XANES analysis (Pratesi *et al.*, 2012 ▸). An involvement of axial hydroxide in Cu^III^ binding leading to a stability in the coordination but a slight distortion of the Cu–N geometry was reported (Kaczmarek *et al.*, 2005 ▸). Hence, the refined Cu^II^Aβ_4–8/12/16_ model – first-shell Cu coordination in the ATCUN-binding site with an arrangement of four nitrogen ligands in equatorial positions, including the phenylalanine amino group N(Phe4); two deprotonated amides from the first two peptide bonds, N(Arg5) and N(His6); and an N atom of the imidazole side chain of the histidine residue, ND1(His6), with a fifth coordination oxygen along the tetragonal pyramid geometry – was initially used for Cu^III^Aβ data fitting. Room-temperature Cu^III^:Aβ EXAFS measurements at oxidative potentials of 0.95 and 1.05 V were investigated.

The refined Cu^II^:Aβ model was individually fit to four XAS-EC Cu^III^:Aβ_4–16(1)_ and Cu^III^:Aβ_4–12(1, 2, 3)_ datasets. Our refinement model allowed individual fitting of 13 structural parameters including ten radial distances, the overall scaling 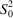 , the energy-threshold offset δ*E*_0_ and one independent thermal parameter for the axial water σ^2^, out to a single scattering path length up to 5 Å, with chemical-bond restraints. Seven restraint functions are used to maintain reasonable parameters for 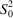 , σ^2^ and five bond-length estimates. Three thermal parameters for the nearest-neighbour nitrogens, for the second-, third- and outer-shell neighbours, σ^2^, were fixed at 0.001, 0.00105 and 0.011 Å^−2^, respectively. The best fit was obtained for the five-coordinate pyramidal arrangement about the Cu^III^ atom. The improvement of the fit with the addition of an axial water molecule was confirmed by the *F* test. Experimentally calculated *F*_2, 20_ = 9.92 is greater than the tabulated *F*_2, 20, 0.05_ = 3.49 at the significance level of α = 5%. The results for Cu^III^Aβ using *eFEFFit* are given in Table 4 ▸.

**Figure 13:**
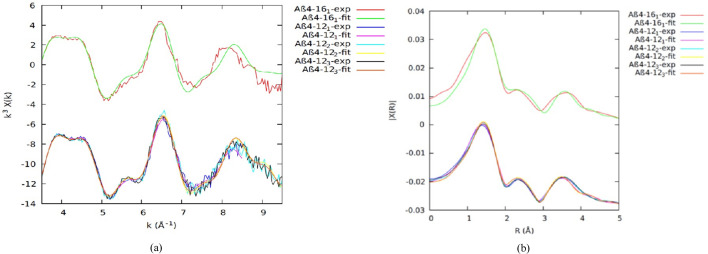
Data fits of the EXAFS for Cu^III^:Aβ_4–12/16_ in (*a*) *k* and (*b*) *r* space using the *eFEFFit* package. Different *k* ranges were used for the refinements: *k* = 3.5–9.5 Å for Cu^III^:Aβ_4–16(1)_ and Cu^III^:Aβ_4–12(2, 3)_, and *k* = 3.5–8.5 Å for Cu^III^:Aβ_4–12(1)_. Each was modelled independently: Fourier transform of Cu^III^:Aβ_4–16(1)_ and Cu^III^:Aβ_4–12(1, 2, 3)_ peptides. Here, *mu2chi* cubic interpolated experimental data have been fitted across *k* = 3.5–9.5 Å.

Refined structural parameters from individual scans are in good agreement within the uncertainties, indicating the consistency of the sample solution throughout the measurement collection. Moreover, the consistency of the derived parameters confirms the absence of radiation damage. The refined distances of Cu^III^ ion for the nitrogen atoms and apical water ranged from 1.84 (2) to 2.1 (1) and 2.11 (2) Å, respectively. These results are mainly consistent with Streltsov *et al.* (2018 ▸) and the literature, but are more robust and accurate with propagated uncertainties. Fig. 13 ▸ shows fitted measurements of the EXAFS for Cu^III^:Aβ_4–16(1)_ and Cu^III^:Aβ_4–12(1, 2, 3)_ in *k* and *R* space.

This investigation of Cu^III^ production corresponds to the Cu^II^ oxidation by H_2_O_2_ that leads to oxidative damage in the peptide (Kaczmarek *et al.*, 2005 ▸; Puri & Edgerton, 2014 ▸). Tay *et al.* (2009 ▸) illustrated the oxidative activity of salivary copper with Hst5 Cu-metal binding. They explicitly discussed the toxicity produced by the oxidation with the presence and absence of H_2_O_2_. Rapid generation of oxidized Cu in Cu^II^ complexes with ascorbic acid and H_2_O_2_ was also reported (Burke *et al.*, 2003 ▸). The reactivity coming from the Fenton mechanism could damage DNA. Similar toxicity could possibly be generated by the Cu^II^ oxidation in Cu:Aβ_4–*y*_ peptides. The water molecule modelled in the low-temperature EXAFS analysis corresponds to the further H_2_O_2_ interaction at an apical arrangement of the copper site (Tsai & Weber, 1992 ▸; Kaczmarek *et al.*, 2005 ▸).

## 7. Supporting information

Detailed *eFEFFit* scripts are provided in the supporting information.

## 8. Conclusions

The XAS-EC setup is a powerful technique for collecting high-quality XAS measurements of biological samples under varying physical conditions. Here, the estimation of uncertainties was obtained from the point-wise variance of the spectra. Radiation damage of the sample was explicitly diagnosed and minimized through the data collection. The consistency of the repeated measurements and refined structural parameters is evidence for the minimal radiation damage. Quantitatively, no damage was observed in the optimized scans reported.

Low-temperature XAS measurements of N-truncated Cu^II^:Aβ_4–8/12/16_ peptides illustrated identical XANES and EXAFS, confirming the consistency of Cu^II^ ATCUN-type binding to four N ligands located in the first three amino acids (FRH). Strong evidence of a fifth ligand in the axial position was observed, yielding a tetragonal pyramidal geometry for the binding site. Neither Tyr10 nor Glu11 contribute to the Cu^II^ binding, and therefore to identical XAS for Cu^II^Aβ_4–8/12/16_. The individual detector-pixel-based variance was used to accurately quantify the uncertainties of the fluorescence measurements. The refined structural parameters were compared by fitting the model with individual and multiple EXAFS from a robust analysis using *eFEFFit* with propagated experimental uncertainties. The XANES and EXAFS analysis strengthens the argument that the identical high-affinity ATCUN-type copper-binding site is located in N-truncated Cu^II^Aβ isoforms detected in Alzheimer’s patients’ brains (Masters *et al.*, 1985*b* ▸).

XAS-EC measurements at room temperature enabled us to investigate the products of the redox process and their structures. Previously reported electrochemical measurements (Mital *et al.*, 2015 ▸) were incapable of demonstrating any response different from the background for Cu^II^:Aβ_4–16_ reduction chemistry, but our XAS-EC measurements provided the reduction details, which are similar to the reduced product of Cu^II^:Aβ_1–16_, at relatively mild potentials. Identification of reduction chemistry for Cu^II^:Aβ_4–8/12_ clearly proves the benefits of XAS-EC experiments for peptides with slow electron-transfer rates. The reduced Cu^I^ ion in Cu:Aβ_4–16_ binds to the *bis*-His site in a quasi-tetrahedral environment geometry. The results and observations of this experiment illustrate that the Cu^II^ and Cu^I^ redox chemistry of Cu:Aβ_4–16_ was driven by a combination of kinetics and thermodynamic effects through the POET reaction pathways explained for Cu^II^:Aβ_1–16_ (Balland & Hureau, 2010 ▸; Streltsov *et al.*, 2018 ▸). N-truncated Aβ peptides have a strong Cu^II^ binding into the high-affinity ATCUN-binding site. However, if a copper ion can access an intermediate-site geometry within the peptide, then, the redox reactions of the copper ion will still feasible. Therefore, the involvement of H13H14 is essential for the Cu^II^:Aβ complexes. Generation of ROS for N-truncated Aβ peptides is different from full-length Aβ peptides due to variations in the kinetics of electron transfer to binding sites. However, involvement of residues through the intermediate sites may precede redox activities in Cu^II^:Aβ complexes. A comprehensive study of intermediate-site structure during reductions can be performed using XAS-EC and the high-energy fluorescence detection (Shearer & Szalai, 2008 ▸; Arrigoni *et al.*, 2018 ▸; Falcone *et al.*, 2023 ▸) approach in future.

XANES features of the oxidative product were observed and structural parameters consistent with crystallographic data were achieved from EXAFS analysis. Cu^II^ and Cu^III^ states bound to a five-coordination ATCUN-type copper-binding site including an axial oxygen from a water molecule exit in oxidation of Cu^II^:Aβ_4–12/16_ complexes. The redox process includes the Cu^II^ catalyzed oxidation of residues. The current EXAFS analysis does not reveal the redox state of ligands not bound to Cu. Further investigations can be conducted for different Cu:Aβ sequences to explore redox behaviour and potential structural dependence.

Literature reports the evidence of using N-truncated Aβ resonant for better drug targets in the brain than full-length Aβ peptides (Bayer & Wirths, 2016 ▸). A comprehensive knowledge of the redox behaviour and atomic structure of Cu-bound N-truncated Aβ complexes would be beneficial in developing Aβ-related therapies and diagnostic processes, and identifying the functionality of different Aβ sequences. A similar behaviour of ATCUN-bound Cu^II^:Aβ_4–*y*_ was observed in transmembrane Cu transport protein (CTR1). High-affinity ATCUN-bound complexes including the *bis*-His binding motif (oligomers Aβ_4–*y*_) are separated in CTR1, allowing Cu^II^ reduction to Cu^I^, which follows further chemistry of generating ROS. Monomeric Aβ_4–*y*_ acts as an associate for CTR1 to transfer copper.

Moreover, the determined structural and chemical properties of Cu:Aβ_4–*y*_ are comparable to functions of AMPs such as Hst5 including ATCUN and the *bis*-His motif. AMPs follow mechanisms of intracellular killing resulting in the inhibition of mitochondrial respiration, possibly via ROS and oxidation stress produced locally and temporarily (Kaczmarek *et al.*, 2005 ▸). Hst5 with copper in ascorbic reductant produces noticeable amounts of hydrogen peroxide, H_2_O_2_ (Houghton & Nicholas, 2009 ▸). Redox reactions of Cu^II^ complexes with an ATCUN site (Pratesi *et al.*, 2012 ▸; Jin & Cowan, 2005 ▸) provide details of generating hydroxyl-like radicals and associate with the production of complexes accommodating Cu^III^ in a vigorous oxidative environment. N-truncated Cu:Aβ_4–*y*_ consists of an ATCUN-binding site and may have a similar Hst5-type mechanism of antimicrobial activity and perform as an effector molecule in the innate immune system. This could be a supplementary function of antimicrobial activity to form transmembrane pore and membrane binding (Brogden, 2005 ▸; Soscia *et al.*, 2010 ▸).

Overall, our experiments illustrate that XAS can characterize the electronic and molecular structure of an absorber in a biological metalloprotein sequence with accurate experimental measurements and propagated experimental uncertainties. As mentioned above, the experimental uncertainties of the measurements were obtained from the point-wise variance of the spectra. Standard errors of fluorescence measurements were used as uncertainties for fitting. Use of calculated uncertainties rather than using an estimated uncertainty enables a reliable quantification for the structural refinements of sample fits. The current work determined uncertainties in XAS analysis software packages for structural fitting. The structural information of N-truncated peptides is sufficient to understand their metal chemistry. This work demonstrates the process of collecting XAS measurements of metalloprotein samples of smaller quantities (<1 ml, 1 m*M*) under an electrochemical environment. The quality of measurements is sufficient for structural analysis. Moreover, the current work demonstrates insight into the redox-reaction paths achieved from XAS-EC and the possibility of accommodating unstable coordinating sites in catalytic reactions of peptides. These experiments reveal a profound form of redox behaviour that is important in the chemistry of Cu:Aβ aggregates. This leads to a final question (hypothesis?): is it necessary for the development of Alzheimer’s dementia to have N-truncated Aβ_4–*y*_ peptide fragments in the brain, rather than the more weakly binding and less reactive Aβ_1–*y*_ peptide fragments or the largely inactive Aβ_1–42_ peptide possibly leading to the plaque? Furthermore, is it also necessary for the development of Alzheimer’s dementia to have peptide fragments in the brain with *y* > 16 (these exist) so that reactive oxidation damage can occur? 

## Supplementary Material

Script to run eFEFFIT for the Cu(I)-binding model. DOI: 10.1107/S2052252524001830/oz5005sup1.txt

Script to run eFEFFIT for the Cu(II)-binding model. DOI: 10.1107/S2052252524001830/oz5005sup2.txt

The low-temperature (5K-10K) dead-time corrected, defective-pixel corrected and normalized for dispersion I_f/I_0 measurements of Cu(II):amyloid-beta(4-12/8), and their uncertainties for mu2chi. DOI: 10.1107/S2052252524001830/oz5005sup3.txt

The low-temperature dead-time corrected, defective-pixel corrected and normalized for dispersion I_f/I_0 measurements of Cu(II):amyloid-beta(4-16), and their uncertainties for mu2chi. DOI: 10.1107/S2052252524001830/oz5005sup4.txt

The room-temperature dead-time corrected, defective-pixel corrected and normalized for dispersion I_f/I_0 measurements of Cu(I):amyloid-beta(4-16), and their uncertainties for mu2chi. DOI: 10.1107/S2052252524001830/oz5005sup5.txt

The room-temperature dead-time corrected, defective-pixel corrected and normalized for dispersion I_f/I_0 measurements of N-truncated Cu(III):amyloid-beta peptides. DOI: 10.1107/S2052252524001830/oz5005sup6.txt

## Figures and Tables

**Figure 14:**
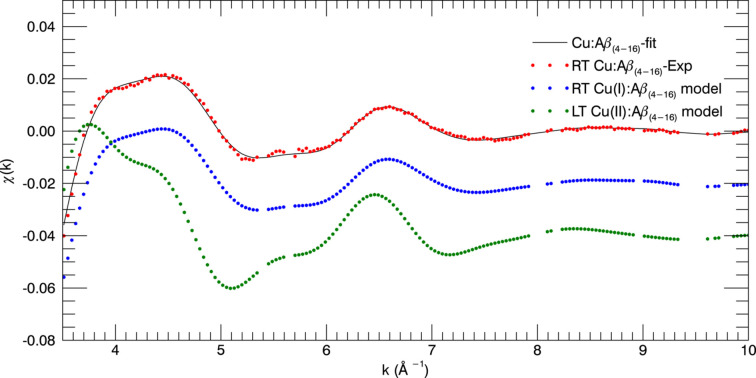
LCA of the experimentally collected Cu:Aβ_4–16_ XAS room-temperature (RT) data using refined Cu^I^:Aβ_4–16_ XAS room-temperature data and refined Cu^II^:Aβ_4–16_ XAS low-temperature data as the fitting standards.

**Table 1:** Best fit of the low-temperature Cu^II^:Aβ_4–8/12/16_ EXAFS using *eFEFFit* Individual scans returned the same parameter values confirming an ATCUN site without pleomorphism. The standard errors from least squares are given in parentheses. χ^2^ and χ^2^ reduced, 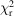 , values are provided for ease of recognizing significance. Propagated uncertainties and high-accuracy experimental data were used in structural refinements.

Individual scan	Aβ_4–16(1)_	Aβ_4–16(2)_	Aβ_4–16(3)_	Aβ_4–12_	Aβ_4–8(1)_	Aβ_4–8(2)_	Aβ_4–8(3)_
*N*_idp_	246	246	246	192	223	221	222
*N*_pars_	14	14	14	14	14	14	14
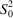	0.98 (3)	0.9999 (8)	0.9998 (8)	1.0000 (9)	1.0000 (7)	1.0000 (5)	1.0000 (6)
δ*E*_0_ (eV)†	3.5 (3)	3.4 (3)	3.4 (3)	3.3 (3)	4.2 (3)	4.3 (2)	3.9 (3)
							
*r*-N (His6) (Å)	1.855 (4)	1.835 (6)	1.831 (8)	1.847 (3)	1.860 (6)	1.858 (2)	1.834 (8)
*r*-N d1 (His6) (Å)	1.96 (2)	1.960 (9)	1.95 (1)	1.98 (3)	1.940 (6)	1.972 (6)	1.967 (6)
*r*-N (Arg5) (Å)	1.96 (2)	1.9635 (2)	1.9604 (2)	1.95 (2)	1.9825 (2)	1.9696 (1)	1.9617 (1)
*r*-N (Phe4) (Å)	2.057 (7)	2.03 (1)	2.05 (1)	2.05 (1)	2.053 (8)	2.046 (6)	2.00 (2)
							
*r*-O d1-d (Å)	3.02 (2)	3.00 (3)	3.00 (2)	3.01 (3)	3.02 (3)	3.02 (2)	3.00 (2)
*r*-C (Phe4) (Å)	2.85 (1)	2.77 (2)	2.77 (2)	2.84 (2)	2.79 (1)	2.78 (1)	2.77 (2)
*r*-C a (Arg5) (Å)	2.78 (1)	2.84 (1)	2.83 (1)	2.78 (2)	2.84 (2)	2.86 (1)	2.84 (2)
*r*-C g (His6) (Å)	2.99 (2)	2.93 (3)	2.93 (2)	3.01 (1)	2.95 (2)	2.93 (2)	2.97 (1)
*r*-C e1 (His6) (Å)	2.94 (2)	2.96 (3)	2.95 (2)	2.95 (2)	2.99 (2)	2.97 (2)	2.94 (2)
*r*-O (water), (Å)	2.08 (4)	2.08 (2)	2.04 (3)	2.09 (3)	2.12 (2)	2.13 (2)	2.10 (1)
							
σ^2^ (Å^2^)‡	0.007 (2)	0.0084 (8)	0.0089 (7)	0.006 (2)	0.010 (1)	0.0083 (8)	0.0073 (7)
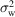 (Å^2^)§	0.0201 (8)	0.014 (8)	0.015 (7)	0.0200 (9)	0.0201 (7)	0.0200 (5)	0.012 (5)
							
χ^2^ with O	145.5	118.3	120.1	125	93.8	42.7	65.3
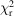 with O	0.69	0.56	0.57	0.81	0.51	0.23	0.36
χ^2^ without O	166	135	142.9	133.2	96.3	45.5	88.9
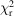 without O	0.78	0.64	0.67	0.85	0.52	0.25	0.48
δχ^2^	20.4	16.7	22.8	8	2.6	2.8	23.6
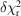 (%)	11.5	11.6	15.2	4.8	1.6	5.1	25.8

† δ*E*_0_ is the refined offset of the photoelectron threshold.

‡ Isotropic thermal parameters for the first- and second-shell N and O atoms were fixed to 0.001 and 0.00105 Å^2^, respectively, in consonance with general fits and physically meaningful ranges, after considering several refinements of each scan. This row contains the fitted thermal parameter for the third and outer shells.

§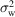 is the thermal isotropic parameter for the water molecule.

**Table 2:** Multiple-scan fit results of the low-temperature Cu^II^:Aβ EXAFS using *eFEFFIT*

Scans	Aβ_4–16(1, 2, 3)_	Aβ_4–8(1, 2, 3)_	Aβ_4–8/12/16_
*N*_scans_	3	3	7
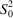	0.97 (5)	1.0000 (4)	1.0000 (6)
δ*E*_0_ (eV)	3.5 (3)	4.0 (2)	3.6 (2)
			
*r*-N (His6) (Å)	1.843 (5)	1.850 (2)	1.845 (6)
*r*-N d1 (His6) (Å)	1.95 (1)	1.964 (3)	1.952 (6)
*r*-N (Arg5) (Å)	1.97 (2)	1.96110 (8)	1.9704 (1)
*r*-N (Phe4) (Å)	2.04 (1)	2.040 (4)	2.04 (1)
			
*r*-O d1-d (Å)	3.01 (1)	3.01 (1)	3.01 (1)
*r*-C (Phe4) (Å)	2.78 (1)	2.773 (8)	2.78 (1)
*r*-C a (Arg5) (Å)	2.83 (1)	2.841 (9)	2.838 (7)
*r*-C g (His6) (Å)	2.94 (2)	2.96 (1)	2.94 (1)
*r*-C e1 (His6) (Å)	2.96 (3)	2.95 (1)	2.97 (2)
*r*-O (water) (Å)	2.06 (4)	2.11 (1)	2.08 (1)
			
σ^2^ (Å^2^)†	0.009 (1)	0.0083 (5)	0.0089 (4)
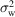 (Å^2^)	0.016 (5)	0.0200 (4)	0.019 (7)
			
χ^2^ with O	401.9	219.4	600.6
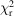 with O	0.61	0.38	1.04
χ^2^ without O	451.4	238.8	678.5
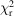 without O	0.68	0.41	1.17
δχ^2^	49.5	19.4	77.9
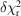 (%)	10.7	7.8	11.4

† Isotropic thermal parameters for the first and second shells were fixed to 0.001 and 0.00105 Å^2^, respectively, in consonance with general fits and physically meaningful ranges, after considering several refinements of each scan. This row represents thermal parameters for the third and outer shells.

**Table 3:** Best fit of EXAFS data during electrolysis of room-temperature Aβ fragments using *eFEFFit* χ^2^ and 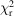 for structures with water and without water are compared. The structures with water give significantly better χ^2^: the Cu^I^ is bound to the *bis*-His site with an axial water in all Aβ_4–16_ peptide fragments. Independent distances to dominant nearest-neighbour atoms and peptides are given. The fourth column shows the results of multi-data refinement of Cu^I^Aβ_4–16(1, 2)_ datasets.

Complex	Cu^I^Aβ_4–16(1)_	Cu^I^Aβ_4–16(2)_	Cu^I^Aβ_4–16(1, 2)_
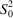	0.85 (4)	0.86 (5)	0.85 (3)
δ*E*_0_ (eV)	5.7 (5)	6.0 (4)	6.0 (3)
*r*-N (His13) (Å)	1.816 (8)	1.803 (7)	1.804 (4)
*r*-N (His14) (Å)	1.98 (1)	1.94 (1)	1.942 (8)
*r*-O (His13) (Å)	1.935 (5)	1.94 (1)	1.940 (7)
*r*-O (water) (Å)	2.24 (1)	2.25 (2)	2.25 (1)
N-σ^2^ (Å^2^)†	0.0009 (4)	0.0019 (6)	0.0018 (4)
O-σ^2^ (Å^2^)†	0.0010 (7)	0.0010 (5)	0.0010 (2)
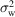 (Å^2^)	0.014 (2)	0.018 (2)	0.018 (1)
χ^2^ with O	20.7	334.2	441.90
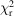 with O	0.14	2.14	1.39
χ^2^ without O	78.5	912.6	1070
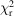 without O	0.51	5.76	3.34
δχ^2^	57.7	578.4	628
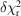 (%)	73.21	62.91	58.38

† Thermal parameter for the first-shell N and O atoms. For the higher shells, 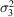 (*r* > 3.0 Å) = 2σ^2^ and 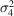 (*r* > 4.0 Å) = 2.5σ^2^. The fit without the O (water) ligand was compared with the best fit. Calculated and tabulated *F*-test values at 5% significance level for the models with and without the O (water) ligand are reported.

**Table 4:** Best fit of the low-temperature (Cu^III^) Aβ_4–12/16_ EXAFS using *eFEFFit* Estimated standard errors from the fit are given in parentheses. Isotropic thermal parameters for the first-shell N, for the second shell, and for the third- and outer-shell neighbours are fixed to 0.001, 0.00105 and 0.011 Å, respectively, after considering several refinements for each scan. χ^2^ and 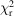 for structures with water and without water are compared. The structures with water give significantly improved χ^2^, demonstrating that the Cu is bound to the ATCUN site with an axial water consistently in all three Aβ peptide fragments. Independent distances to the dominant nearest-neighbour atoms and peptides are given. The sixth column shows the results of multiple-dataset refinement of all Aβ_4–16/12_ datasets at room temperature. Whilst broadly consistent, there is perhaps some evidence that Aβ_4–16(1)_ is reporting different parameters, and note that the data are not equally definitive.

Fragment scan	Aβ_4–16(1)_	Aβ_4–12(1)_	Aβ_4–12(2)_	Aβ_4–12(3)_	Aβ_4–16/12_
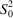	1.0000 (2)	1.0 (1)	0.84 (3)	0.88 (2)	0.88 (2)
δ*E*_0_ (eV)	2.5 (4)	5.0 (4)	5.1 (3)	5.2 (3)	4.9 (3)
*r*-N (Arg5) (Å)	1.84 (2)	1.98 (6)	1.95 (1)	1.9545 (5)	1.93 (2)
*r*-N (His6) (Å)	1.940 (1)	1.86 (4)	1.861 (3)	1.85 (1)	1.86 (2)
*r*-N d1 (His6) (Å)	2.03 (2)	1.92 (2)	1.9908 (3)	2.0069 (2)	2.0033 (2)
*r*-N (Phe4) (Å)	2.00 (2)	2.1 (1)	2.01 (2)	1.99 (2)	2.01 (3)
					
*r*-O d1-d (Å)	3.09 (5)	3.05 (5)	3.02 (3)	3.02 (3)	3.02 (2)
*r*-C (Phe4) (Å)	2.788 (8)	2.90 (4)	2.790 (7)	2.793 (6)	2.789 (4)
*r*-C a (Arg5) (Å)	2.89 (2)	2.83 (3)	2.88 (2)	2.88 (1)	2.88 (1)
*r*-C g (His6) (Å)	3.00 (6)	2.85 (2)	2.92 (2)	2.92 (2)	2.93 (2)
*r*-C e1 (His6) (Å)	2.99 (3)	2.982 (5)	2.97 (2)	2.99 (1)	2.983 (9)
*r*-O (water) (Å)	2.08 (2)	2.10 (6)	2.08 (2)	2.11 (2)	2.09 (2)
					
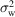 (Å^2^)	0.015 (6)	0.01 (3)	0.019 (5)	0.2001 (6)	0.019 (9)
χ^2^ with O	5.0	67.8	94.2	57.6	180.4
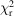 with O	0.04	0.58	0.65	0.40	0.62
χ^2^ without O	41.1	121.6	103.8	67.6	225.0
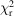 without O	0.29	1.02	0.70	0.46	0.77
δχ^2^	36.1	53.7	9.6	10.0	45
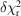 (%)	87.2	43.3	8.0	13.6	19.5

## Appendix A Linear combination analysis for calculation of Cu^I^:Cu^II^ ratio in room-temperature data

LCA was performed to explore if room-temperature XAS data could be identified as a mixture of Cu^I^ and Cu^II^. The LCA standards included refined Cu^I^:Aβ_4–16_ XAS room-temperature data and refined Cu^II^:Aβ_4–16_ XAS low-temperature data. Experimentally collected Cu:Aβ_4–16_ XAS-EC data were fitted as

where *A* is the percentage contribution of Cu^I^ in the hypothesized mixture. This LCA is based on the EXAFS *k* space from 3.5 to 10 Å^−1^. The fitting returned a Cu^I^:Cu^II^ ratio of 0.994:0.006 in the room-temperature mixture (Fig. 14 ▸). No significant Cu^II^ contamination exists in the room-temperature mixture, especially given the 1.44% uncertainty of the LCA ratio. The standards used here are from the experiments at different conditions (Cu^II^:Aβ_4–16_ XAS, different temperatures and under no voltage). The LCA can be further investigated by conducting further experiments. Whilst this LCA is not foolproof, it is strongly suggestive of complete reduction.

Streltsov *et al.* (2008 ▸) performed linear combination fitting (LCF) to Cu^I^:Aβ_4–16_ XAS-EC data using the *ATHENA* package. They found a Cu^I^:Cu^II^ ratio of 97:3%. However, the analysis was conducted using previous unpublished Cu^I^:Aβ_1–16_ data as the standard. We anticipate that it would have be more transparent if the experimental data under similar conditions had been used for the LCF. Our standards have these similar experimental conditions and therefore our Cu^I^:Cu^II^ ratio is more reliable.
